# Bringing the Nonlinearity of the Movement System to Gestural Theories of Language Use: Multifractal Structure of Spoken English Supports the Compensation for Coarticulation in Human Speech Perception

**DOI:** 10.3389/fphys.2018.01152

**Published:** 2018-09-03

**Authors:** Rachel M. Ward, Damian G. Kelty-Stephen

**Affiliations:** Department of Psychology, Grinnell College, Grinnell, IA, United States

**Keywords:** speech perception, phoneme, coarticulation, multifractal, mouse-tracking

## Abstract

Coarticulation is the tendency for speech vocalization and articulation even at the phonemic level to change with context, and compensation for coarticulation (CfC) reflects the striking human ability to perceive phonemic stability despite this variability. A current controversy centers on whether CfC depends on contrast between formants of a speech-signal spectrogram—specifically, contrast between offset formants concluding context stimuli and onset formants opening the target sound—or on speech-sound variability specific to the coordinative movement of speech articulators (e.g., vocal folds, postural muscles, lips, tongues). This manuscript aims to encode that coordinative-movement context in terms of speech-signal multifractal structure and to determine whether speech's multifractal structure might explain the crucial gestural support for any proposed spectral contrast. We asked human participants to categorize individual target stimuli drawn from an 11-step [ga]-to-[da] continuum as either phonemes “GA” or “DA.” Three groups each heard a specific-type context stimulus preceding target stimuli: either real-speech [al] or [a], sine-wave tones at the third-formant offset frequency of either [al] or [aɹ], and either simulated-speech contexts [al] or [aɹ]. Here, simulating speech contexts involved randomizing the sequence of relatively homogeneous pitch periods within vowel-sound [a] of each [al] and [aɹ]. Crucially, simulated-speech contexts had the same offset and extremely similar vowel formants as and, to additional naïve participants, sounded identical to real-speech contexts. However, randomization distorted original speech-context multifractality, and effects of spectral contrast following speech only appeared after regression modeling of trial-by-trial “GA” judgments controlled for context-stimulus multifractality. Furthermore, simulated-speech contexts elicited faster responses (like tone contexts do) and weakened known biases in CfC, suggesting that spectral contrast depends on the nonlinear interactions across multiple scales that articulatory gestures express through the speech signal. Traditional mouse-tracking behaviors measured as participants moved their computer-mouse cursor to register their “GA”-or-“DA” decisions with mouse-clicks suggest that listening to speech leads the movement system to resonate with the multifractality of context stimuli. We interpret these results as shedding light on a new multifractal terrain upon which to found a better understanding in which movement systems play an important role in shaping how speech perception makes use of acoustic information.

## Introduction

Language use is one of the more challenging accomplishments of human behavior for scientists to explain (Chomsky, 1959). Curiously, it becomes no less challenging as we attempt to break spoken language usage into its smallest bits. For instance, even at the fine scale of phonemes pairing individual vowels with individual consonants, speech modeling continues to the present day to struggle with “coarticulation” (Hill et al., 2017), i.e., that the physiological production of each phoneme changes depending on its context in the speech stream. Human listeners manage to compensate for coarticulation to the point of responding systematically to audible changes with context—even without explicitly noticing the context effects and even, instead, with having the experience of stable phonemic recognition (Zamuner et al., 2016; Viswanathan and Kelty-Stephen, 2018). To restate, compensation for coarticulation (CfC) is the tendency for human listeners to use the preceding auditory context to perceive ambiguous phonemes. The primary contributions of the present work are twofold. The first proposal is that the listener sensitivity to coarticulatory context effects extends to a finer scale of unnoticed transpositions of the internal sequence within a single vowel. The second proposal is that such transpositions allow us to show that the perceptual compensation for coarticulation depends on the multifractal structure of speech production.

The multifractal questions of the present work immediately follows recent work examining how compensation for coarticulation changed across trials as well as within trials (Viswanathan and Kelty-Stephen, 2018). Participants judged tokens from a [ga]-to-[da] continuum, clicking their choice of [ga] or [da] on a computer screen with a mouse cursor. Generally, participants will compensate for coarticulation by perceiving a “g” sound following an [al] more often than following an [aɹ]. General auditory accounts focus more on the spectral contents of the acoustic signal than on the details of speech production, and they explain the preference for “g” following [al] in terms of contrast between the offsets of the third formant (F3) in [al] and [aɹ] and the onset of F3 in the [ga]-to-[da] token. That is, because the F3 of both [al] and [da] are relatively high compared to the F3 of both [aɹ] and [ga]. The spectral contrast between the high F3 in [al] leads listeners to interpret the F3 of [ga]-to-[da] tokens as having relatively lower frequency (and more “ga”-like) after hearing the high F3 in [al] rather than the low F3 in [aɹ] (Diehl et al., 2004). On the other hand, the gestural account of compensation for coarticulation is that the frontal tongue-tip gesture of [al] pulls forward the ambiguous middle steps of the [ga]-to-[da] continuum into a “ga” sound, but posterior tongue body constriction in [aɹ] does not.

Replicating a tried-and-true experimental paradigm but using longitudinal modeling across the 176 trials opened up a new finding in favor of gestural explanations of compensation for coarticulation (CfC): the effect of spectral contrast in phoneme perception depends on the broader context of a speech sound as opposed to a tone sound. To put this point in greater detail of how participants made speech-perceptual judgments, we can say the following: participants paid progressively less attention to tone contexts as the experiment went on, they exhibited progressively stronger compensation for coarticulation in speech as the experiment went on, and they took more time to process those speech sounds. Specifically, continued experience with speech contexts led participants to make better and more subtle use of the gestural information available in speech rather than in the tones. Meanwhile, tones prompted less attention: participants rapidly learned to tune out information from the tone context and to respond more similarly following the [al] and [aɹ] tone variants alike. Mouse-tracking data provided information about the time-course of the decision-making process on the way to the mouse-click. Stronger use of CfC led to a significant decrease in the number of x-position flips, i.e., the number of times that the mouse-cursor trajectory changes horizontal direction. X-position flips were greatest for the middle steps of the [ga]-to-[da] continuum but decreased across trials in the speech-context condition for slower mouse-tracking movements, which we separate from fast mouse-tracking movements using Calcagnì et al.'s (2017) informational-entropy measures to parse fast movements from the entire trajectory. In summary, results essentially suggested that speech contexts appear to trigger significant change—and improvements—in compensation for coarticulation on multiple time scales.

Multi-scale changes in compensation for coarticulation with speech context invites a new theoretical development that may help explain how speech gestures prove to be so informative for speech perception. Specifically, speech gestures crucial for CfC come from a movement system capable of acting and responding across a wide range of time scales and exhibiting an intermittent, context-sensitive behavior that could only arise from nonlinear interactions across these scales (Kelso et al., 1984; Fowler et al., 2000). Crucially, this nonlinear interactivity is no longer a matter of preference for one theoretical stance or another: nonlinear interactions across time scales is the only competitive explanation for the mounting evidence of intermittent fluctuations following a multifractal sequence appearing throughout the rhythms of the movement system (Ivanov et al., 1998), from the autonomic movements of heartbeats (Ivanov et al., 1999, 2001, 2004; Amaral et al., 2001) to the dynamics of neural signaling (Ivanov et al., 2009), to bipedal gait (Ashkenazy et al., 2002), all the way to more context-sensitive behaviors such as keeping time (Ihlen and Vereijken, 2010), wielding limbs and exploring surfaces (Turvey and Fonseca, 2014) and visually-guided aiming (Carver et al., 2017). By “intermittency,” we mean “unevenness across time.” “Multifractal” refers to a specific pattern of unevenness in which fluctuations grow according to multiple power-laws with fractional—or “fractal” –exponents.

These technical concepts may bring new insights to speech perception. If speech contexts support coarticulation better than symmetrical sine tones, then it is precisely an unevenness across time in speech that carries perceptual information to the listener. If the movement system exhibits fluctuations with multifractal sequence, then perhaps speech contexts bear a multifractal imprint carrying information about the movement system's gestures. Multifractal structure may thus be an important medium through which speech perception can draw on information about articulatory gestures. Hence, a major goal of the present research is to situate gestural theories of speech perception on multifractal foundations.

Because sequence is crucial to the meaning of multifractal evidence, a crucial first step of this attempt to test gestural speech perception is to examine sequence in the production of phonemes. The immediate two questions are: First, does speech production exhibit the intermittency—that is, unevenness across time—consistent with multifractality different from best-fitting linear models? And second, does the multifractal sequence of phoneme production impact phoneme perception? Multifractal analysis is essentially a statistical method for using sequence to test whether nonlinear mechanisms generate intermittent events. If gestures provide support for CfC, and if we want to entertain the idea that multifractality is an important part of the movement systems generating that gestural support, then an experimental test of the multifractal foundation should begin by destroying the original order.

In this original research, we open possibility of multifractal structure for gestural support by replicating the traditional [ga]/[da] phoneme-categorization task with one small change: in addition to presenting tone and speech contexts, we also introduce a simulated-speech context. The simulated-speech context will have the same spectral contrasts but none of the same sequence that human speech-articulatory apparatus would produce. We will produce simulated-speech contexts by randomizing the sequence of relatively homogeneous, repeating segments of open vocalization (i.e., called “pitch periods”) in the audio waveform, thus preserving the sounds' own category membership but destroying its fine-grained sequence. If sequence does not matter to speech perception, and if only spectral contrast matters, then the simulated-speech context should have no effect on CfC (i.e., no effect on prompting “ga” responses after [al] rather than [aɹ]). However, if sequence in the audio waveform does matter for the use of speech contexts in CfC, then we make two general predictions. Our first prediction was that the CfC effects following simulated-speech contexts should resemble CfC effects following tone contexts, and our second prediction was that multifractal estimates of the context's audio waveform should predict the differences in response to the different contexts.

It is important to emphasize that the sequence-destruction that produced simulated-speech contexts was extremely subtle—so subtle as to be undetectable by participants. Randomizations of sequence that leaves original formants relatively unchanged leaves speech perception unchanged as well (Saberi and Perrott, 1999; Lachs and Pisoni, 2004), and we can leave the ends of the formants (i.e., the [l] and [ɹ]) determining spectral contrast completely intact. The intriguing potential is whether leaving these formant offsets intact but randomizing the [a] vowels preceding those offsets could undermine the gestural basis of the speech context. Such simulated-speech contexts would pose participants an intriguing challenge: with formants half approximated and half intact, participants need not be consciously aware of any difference in the category membership of context phonemes, but they will be experiencing the sounds through a contrived sequence that would only give the impression of apparent gestures that might have generated the sounds. If we find a difference by scrambling the sequence of the [a] vowel in [al] and in [aɹ], then the multifractal structure of the speech gestures may be a crucial background support for spectral contrasts.

Randomizing the sequence offers a clear comparison between two possible options. If spectral contrasts matter but gestural structure does not, then destroying the sequence by randomizing within homogeneous parts of the formants should produce no effect on speech perception. On the other hand, if randomizing homogeneous parts of the formants but leaving formant ends intact has any effect on speech perception, then it may be compelling evidence for elaborating the gestural account of speech perception. The present work may thus be the latest in showing how gestural information can indeed complement general auditory models of speech perception (Laurent et al., 2017; Mitra et al., 2017).

This proposal to delicately destroy the sequence of speech sounds might serve to situate the gestural account of language perception atop a multifractal foundation. If the formants remain similar in their homogeneous portions and if formant ends remain identical, then the average temporal structure should be the same. In that case, it is worth noting two points at this juncture. First, multifractal structure will change with order even when average temporal structure remains the same, and so that very statistical property that we noted above as key for expressing interactions across scales could help us to differentiate specific randomized variants. Second, variation in fractal structure of speech signals has lately emerged as one of few effective components in state-of-the-art machine-learning algorithms aimed at using voice-analysis to predict the onset of Parkinson's disorder (Tsanas et al., 2011).

Multifractality is a multifaceted mathematical construct, and we can point to two multifractal estimates, namely, W_MF_ and t_MF_. For any individual series of changes in acoustic pressure, we can estimate not just the multifractality of that series (w_MF_) but also the t-statistic comparing original-series multifractality to multifractality for the best-fitting linear models of the original series. The “w” here stands for “width” of a multifractal spectrum. In the interest of deferring technical detail to the section Multifractal Analysis, we only note here that multifractality is greater as the multifractal spectrum is wider. In a rough intuitive sense, this spectrum width expresses variability in a way that does not presume homogeneous variation—standard deviation is a much more widely known measure of variability, but it presumes that variation is identically and independently distributed. So, w_MF_ is essentially a variability measure made for intermittent signals. On the other hand, t_MF_ indicates the degree to which multifractality differs from what could be expected from linearity. Multifractality (w_MF_) and the nonlinearity it entails (t_MF_) are both important for the movement system (Ihlen and Vereijken, 2010; Turvey and Fonseca, 2014; Carver et al., 2017).

The main idea of this research is to investigate whether the multifractality (w_MF_) and the nonlinearity (t_MF_) in speech-production is an important support for speech perception using speech contexts. What we aim to test is a twofold foundation for gestural approaches to language use: first, if gestures generating speech are multifractal, then the listener may show changes in perceptual responses sensitive to multifractal fluctuations in audio waveforms of speech, and second, listeners may benefit from having a movement system ready to carry these multifractal fluctuations forward into behaviors responding to speech.

The present work documents a replication of the earlier design (Viswanathan and Kelty-Stephen, 2018), using real-speech and tone contexts with the addition of simulated-speech contexts, as well as using mouse-tracking to capture the mouse-cursor trajectory on the way to clicking the perceptual response on the screen. Multifractal analysis and regression models using multifractal estimates served to quantify how much the nonlinearity of each of these context sounds contributes to the judgment of the target phoneme presented after the context. Important for the test of whether multifractal structure matters will be speech that sounds identical to human listeners despite actually differing in sequence. The simulated-speech context stimuli is absolutely essential for our test of multifractal effects on compensation for coarticulation. Without simulated-speech contexts, any difference in multifractality would be reducible to and indistinguishable from differences in context (real speech vs. tone) or in frequency ([al] vs. [aɹ]). It is only with the inclusion of simulated-speech contexts that we might have tokens of ([al] vs. [aɹ]) that sound like speech but that vary sufficiently in sequence to yield new multifractal estimates.

For the purpose of determining whether speech perception is sensitive to multifractality in speech, we entertain two hypotheses. First, we predict that the simulated-speech contexts lacking the original sequence of the real-speech will show weaker CfC effects, specifically with context sounds leading to weaker marginal reference for “GA” over “DA” following [al] (Hypothesis 1). That is, we predict that effects of simulated speech will resemble those of tones across trials in work by Viswanathan and Kelty-Stephen (2018), showing less difference in “GA response” between [al] and [aɹ] (Hypothesis 1a) and showing faster response (Hypothesis 1b). Second, we hypothesize that the CfC rests on both w_MF_ and t_MF_ as well as on their interaction. That is, we predict that the way human participants judge target speech syllables following a context will depend on the unevenness across time in context sounds, the degree of nonlinearity contributing to that unevenness, and the interaction of these two components; and this difference should appear both in the accumulation of “GA” judgments across the experiments as well as the accumulation of response time in making these judgments (Hypothesis 2).

For the purpose of testing whether human listeners carry multifractal fluctuations in speech contexts forward into behaviors responding to speech, we also had two expectations. The primary expectation for analysis of mouse tracking was that the multifractality of the context-sound stimuli had a demonstrable effect on mouse-tracking behaviors. We modeled the three best-known mouse tracking behaviors (section Traditional geometric mouse-tracking measures), each of which encode aspects of mouse-cursor movements that might signify uncertainty, confusion, or attentional load. Our secondary expectation was that we would replicate the finding by Viswanathan and Kelty-Stephen (2018) that, when accentuating models predicting these class mouse-tracking measures with covariates including information-theoretic entropy-based measures (section Information-theoretic entropy measures of mouse-tracking behaviors) developed by Calcagnì et al. (2017), only one of these classic mouse-tracking behaviors (specifically, x-position flips) would show an increase for the most ambiguous target phonemes, that is, the middle tokens in the [ga]-to-[da] continuum. Our first hypothesis here is that multifractality will contribute to the already known interactions of Context with linear effects of Step, quadratic effects of Step, Block, Trial, Precursor, as well as Calcagnì et al.'s (2017) more recent entropy measures and, in some cases, to entirely replace context in these interactions (Hypothesis 3). Our second hypothesis was that we would only find the main effect of the quadratic effect of [ga]-to-[da] continuum step in x-position flips (Hypothesis 4).

## Methods

### Participants

Forty-two native English-speaking Grinnell College undergraduates who reported normal hearing and corrected or normal vision participated in the study after providing informed consent. This study was carried out in accordance with the recommendations of the Common Rule, Grinnell College Institutional Review Board. The protocol was approved by the the Grinnell College Institutional Review Board. All subjects gave written informed consent in accordance with the Declaration of Helsinki. Experimenters randomly assigned each to one of three groups: speech-context, tone-context, or simulated speech conditions.

### Materials

With the exception of the simulated-speech contexts (section Simulated Speech), all stimulus materials were the same as those used by Viswanathan et al. (2009) as well as Viswanathan and Kelty-Stephen (2018). The simulated-speech contexts were reorderings of these same speech contexts as described below and so had most of the same features.

#### [ga]-To-[da] targets

Participants attempted to correctly categorize tokens from a 11-step continuum of resynthesized consonant-vowel (CV) syllables as either [ga] or [da]. Tokens varied in F3-onset frequency in 100 Hz steps from 1800 to 2,800 Hz, changing linearly to 2,500 Hz steady-state offsets (see Viswanathan et al., 2009). All tokens had identical first, second, and fourth formants, i.e., 500 Hz onset to 800 Hz offset, 1,600 Hz onset to 1,200 Hz offset, and remaining at 3,500 Hz, respectively. Each CV syllable was 215 ms long.

#### Contexts

In all cases, 50 ms of silence separated context and target, but the experimenters randomly assigned each participant to one of three context conditions that determined what sounds participants hear 50 ms before the onset of the target. Table 1 shows the multifractal properties of the contexts in each case.

**Table 1:** Multifractal properties of contexts by type and by precursor, with model-predicted logarithmic-probability of one more “GA” response and model-predicted response time (RT).

			**Model-predicted probability of one more …**	
**Contexts**	***W*_*Mf*_**	***t*_*MF*_**	**“GA” response**	**Second of RT**
**CONTEXT(REALSPEECH)**				
[al]	0.1463	−14.77	91.10%	1.64%
[aɹ]	0.1018	−16.76	68.89%	1.64%
**CONTEXT(TONE)**				
[al]	0.0096	−5.94	71.36%	1.46%
[aɹ]	0.0141	−17.99	37.33%	1.51%
**CONTEXT(SIMULATED SPEECH[SS])**				
[al]	0.1467 to 0.1472	−14.01 to −12.31	91.13–91.49%	1.37–1.46%
[aɹ]	0.1017 to 0.1020	−15.67 to −15.03	70.24–71.27%	1.34–1.38%

*Showing inter-quartile range (i.e., 1st−3rd quartile) for 8 variants of SS context in each precursor*.

##### Real-speech

**Figure 1:**
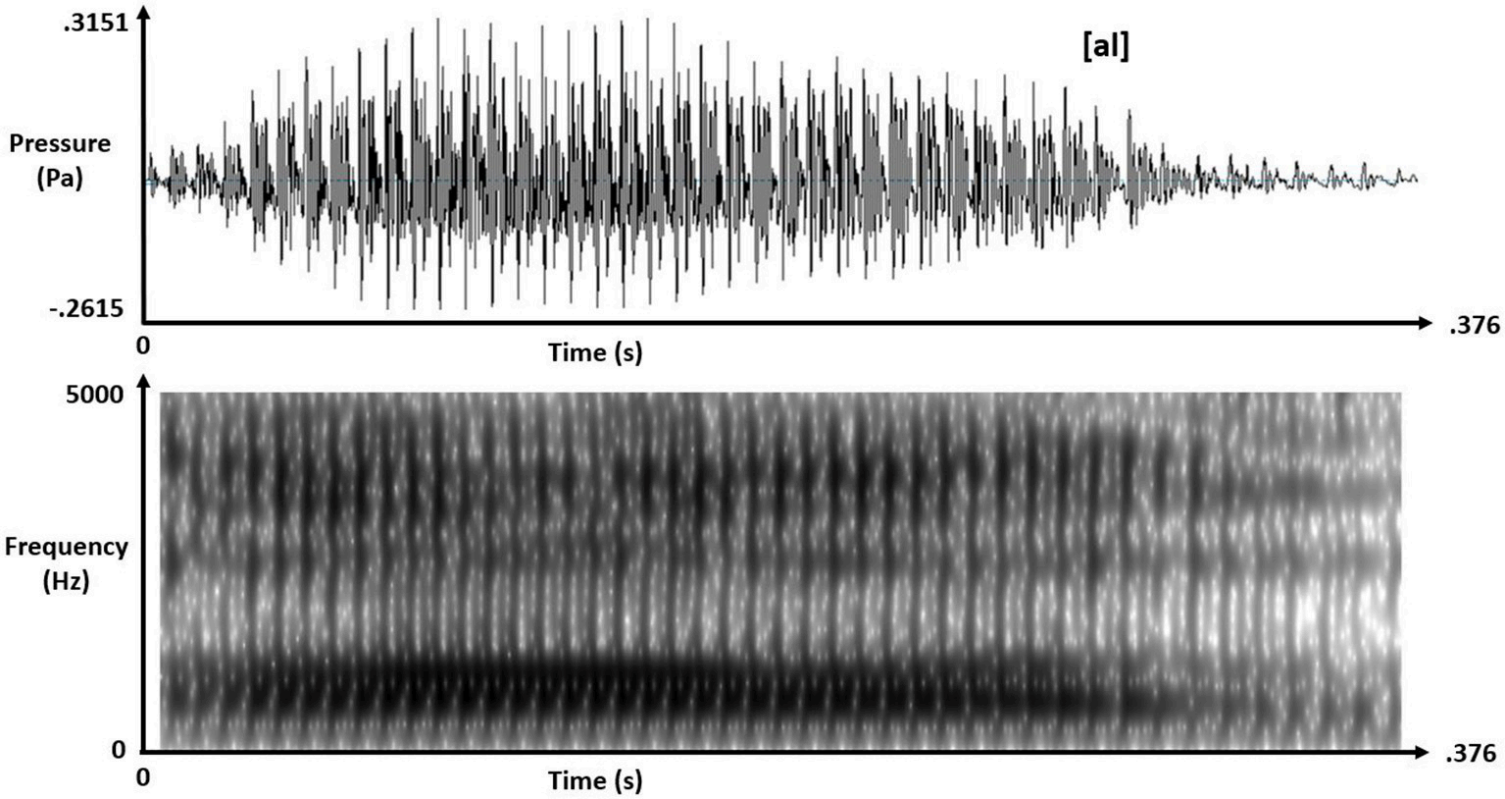
Audio waveform (top) and spectrogram (bottom) for speech context [al].

**Figure 2:**
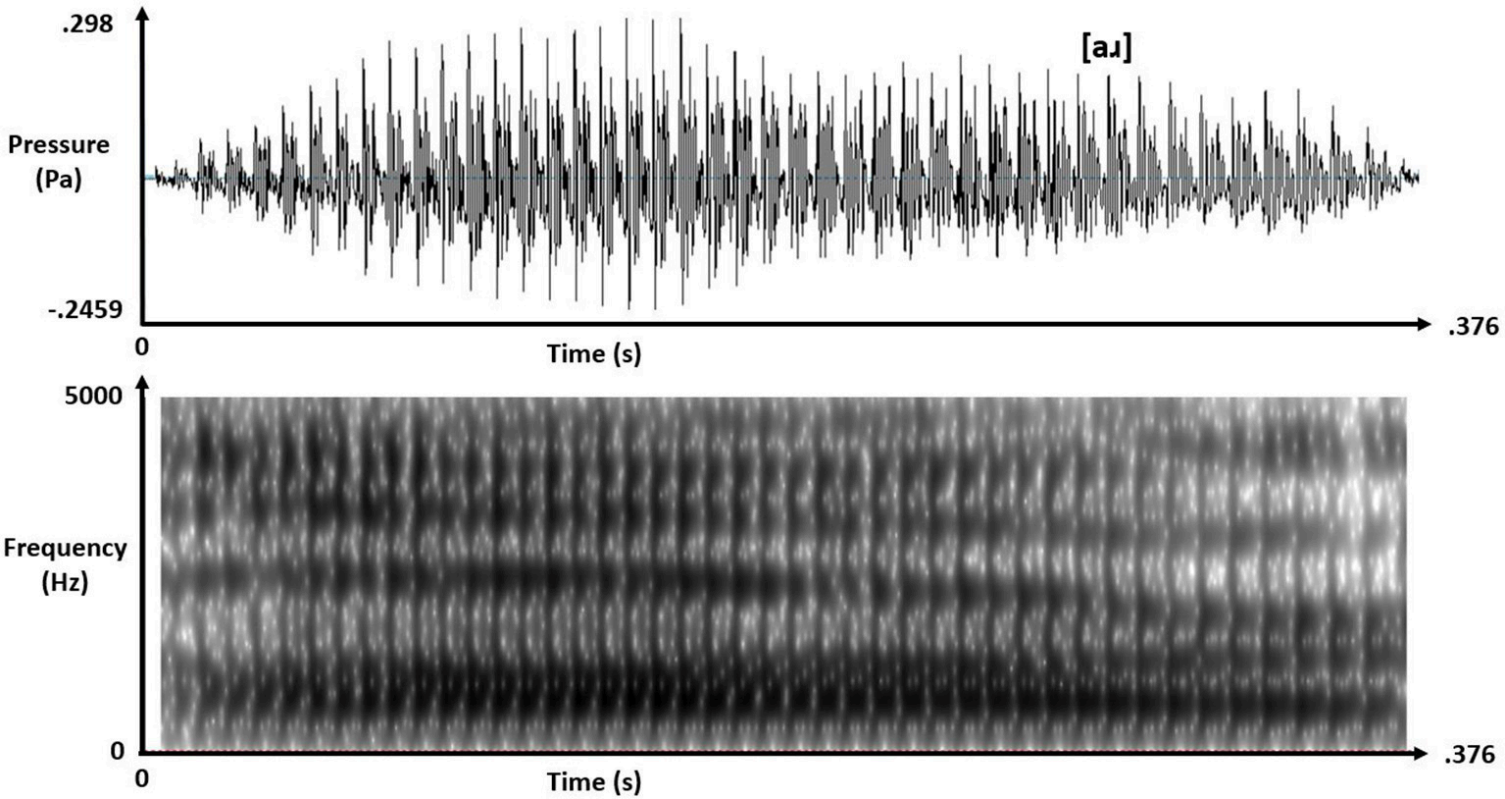
Audio waveform (top) and spectrogram (bottom) for speech context [aɹ].

Real-speech contexts were naturally spoken tokens of [al] and [aɹ] with matching 375-ms duration and intensity but, crucially, differing F3 offsets at approximately 2600 Hz for [al] and 1820 Hz for [aɹ]. Figures 1, 2 depict these real-speech contexts [al] and [aɹ], respectively, both in audio waveforms and in spectrograms.

##### Tones

Tone contexts were duration- and intensity-matched tone analogs of [al] and [aɹ] mimicking the corresponding F3-offset frequencies.

##### Simulated speech

**Figure 3:**
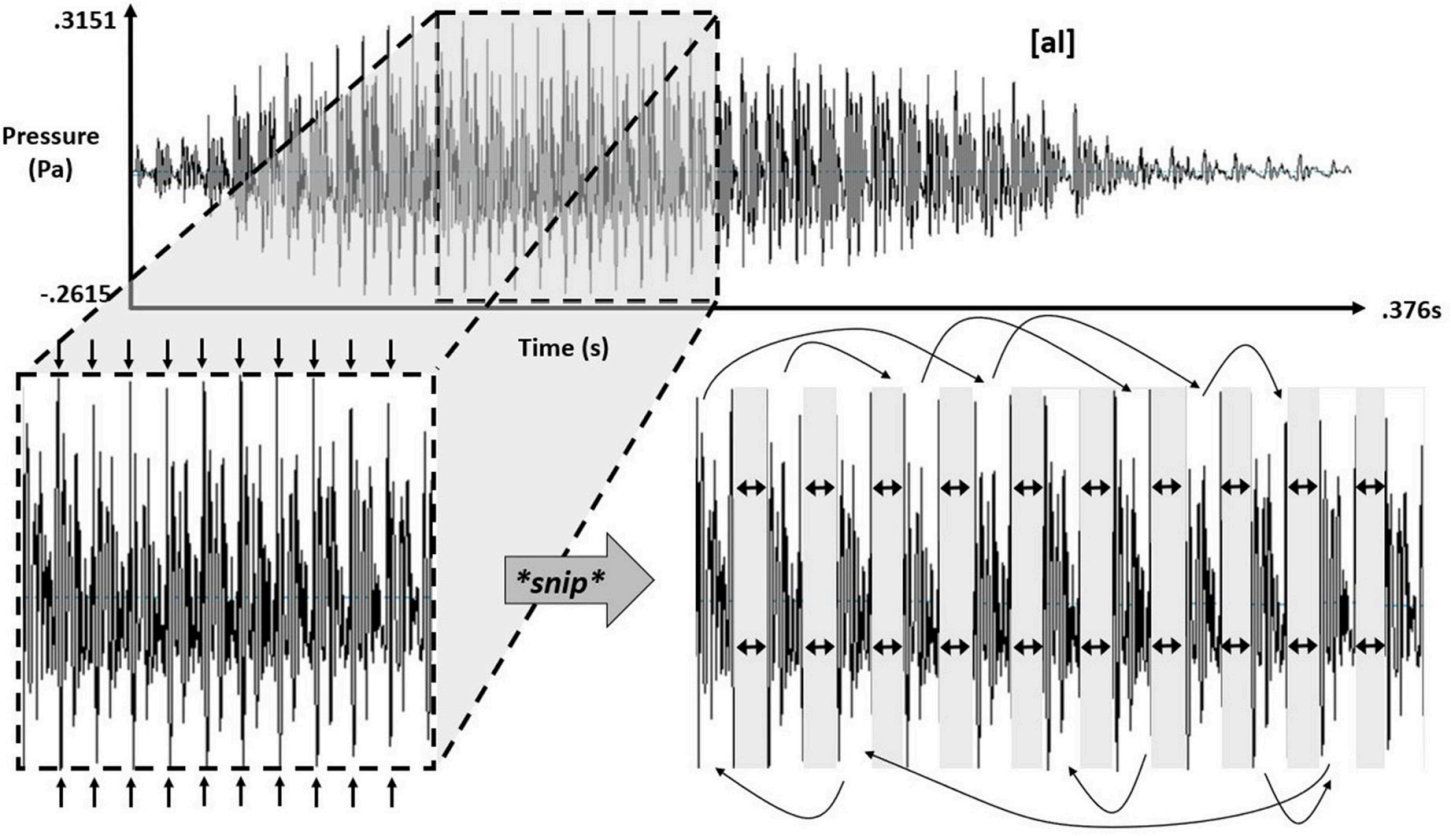
Schematic showing the process of isolating 11 pitch periods within the vowel component of “[al]” and subsequent separation for later randomization, with eight different randomizations each producing a new variant of simulated speech [al].

Simulated-speech contexts were resamplings of the real-speech contexts: for each of the real-speech contexts, experimenters selected 11 similarly shaped oscillations in the digital recording corresponding to the open-voiced “ah” sound before the liquid (i.e., before the /l/ or the /ɹ/), cut them apart and resorted them randomly over the same span of time that they had occurred in the original real-speech recording. Figure 3 schematizes this process for the simulating speech contexts for [al].

**Figure 4:**
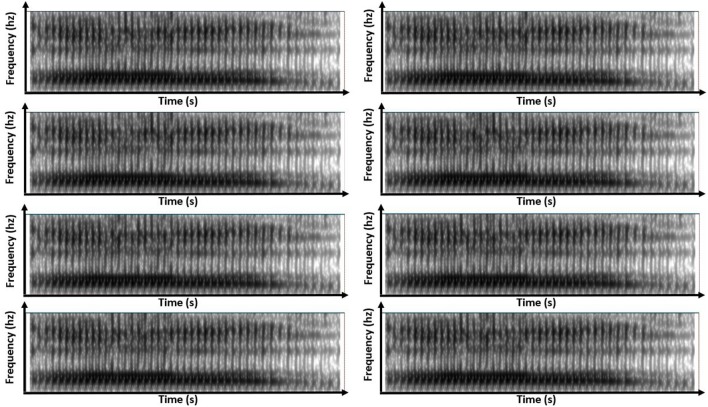
Eight panels of spectrograms for each of the eight variants of simulated-speech contexts simulating the speech context “[al]”.

**Figure 5:**
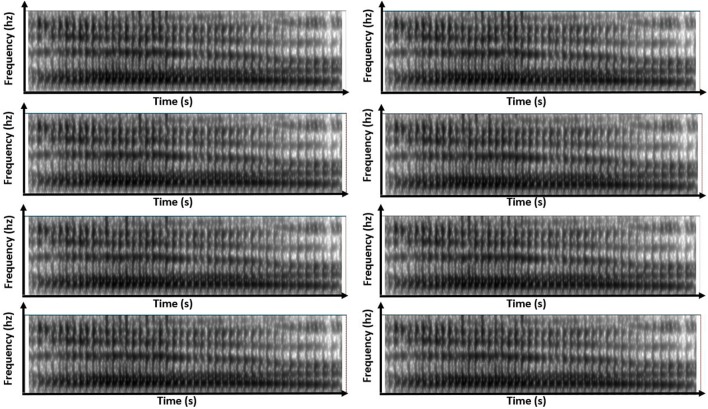
Eight panels of spectrograms for each of the eight variants of simulated-speech contexts simulating the speech context “[aɹ]”.

Simulated-speech contexts were similar to one another. Generally, this resampling of the real-speech contexts generated simulated-speech contexts that resembled the original sounds both in temporal sequence and in spectral structure. Figures 4, 5 show this resemblance visually, depicting the spectrogram for the same eight variants of the simulated-speech [al] and simulated-speech [aɹ] context. A manipulation check confirmed that the real-speech sounds were auditorily indiscriminable from the synthesized-speech sounds: a sample of 10 participants naïve to the manipulation were also unable to distinguish the real-speech contexts from the corresponding simulated-speech context. When asked to identify whether the sound was real speech, these 10 participants identified real-speech contexts as real speech 66% of the time and identified simulated-speech as real speech 73% of the time.

### Procedure

In a two-alternative forced-choice task, participants clicked the computer-mouse cursor over a “Start” box at the screen's lower center to play stimuli. They indicated judgments by clicking either the top-left or top-right of the screen labeled “ga” or “da” (location counterbalanced across participants).

After 10 practice trials presenting only [da] and [ga] endpoints in random order, participants completed 176 trials (16 repetitions of the 11-step stop continuum) judging members of stop continuum in the no-context group. The speech- and tone-context group judged these stops in disyllable sequences and in tone-speech sequences, respectively. The onset of the next trial was controlled by participants who had an option to take breaks between trials. The overall experiment lasted under 30 min. No feedback was provided.

Throughout, on each trial, the presentation software collected data on mouse-tracking behaviors, recording the x-y screen positions of the mouse cursor as participants moved their mouse cursor toward the “GA” icon on the screen that they clicked.

### Analysis

#### Multifractal analysis

Subsequent modeling used Chhabra and Jensen's (1989) [CJ] canonical “direct” algorithm for calculating the multifractal-spectrum width *w*_*MF*_ on the absolute-value of the audio waveform of each context stimuli. This use of absolute-value draws on a long-standing tradition in fractal analyses decomposing signals into magnitudes and signs and using either to quantify nonlinearity (Ashkenazy et al., 2003; Schmitt et al., 2009; Gómez-Extremera et al., 2016). We chose to use only magnitudes using the absolute-value of the audio waveform, but we omitted sign because the symmetry of the audio waveform around zero entailed that the timing of signs would be redundant with magnitude in this case. The absolute value is necessary, and sign series are not usable–both because the CJ method is not defined for non-positive numbers.

This CJ algorithm is preferable to finite-variance scaling methods (e.g., multifractal detrended fluctuation analysis; Kantelhardt et al., 2002) because the time-varying sinusoidal properties of audio waveform constitute a nonstationarity that not even the detrended variance-based methods cannot adequately remove (Bashan et al., 2008). We preferred the direct algorithm to the wavelet transform modulus maxima method as well (Muzy et al., 1991, 1993, 1994) which shows strong agreement with multifractal detrended fluctuation analysis, both in its resulting estimates and in its sensitivity to quasiperiodic trends (Zhou et al., 2013).

The CJ method samples measurement series *u*(*t*) at progressively larger scales, estimating bin-level proportion *P*_*i*_(*L*) within bin *i* of scale *L* is

(1) $$\begin{array}{r} P_{i}\left(L\right)=\;\frac{\sum_{k=\left(i-1\right)L+1}^{iL}u\left(k\right)}{\sum u\left(t\right)} \end{array}$$

CJ method estimates *P*(*L*) for *N*_*L*_ nonoverlapping *L*-sized bins of *u*(*t*) and using parameter *q* to translate them into mass μ_*i*_(*q,L*):

(2) $$\begin{array}{r} \mu_{ij}\left(q,L_{j}\right)=\frac{{\left[P_{ij}\left(L_{j}\right)\right]}^{q}}{\overset{N_{j}}{\underset{i=1}{\sum }}{\left[P_{ij}\left(L_{j}\right)\right]}^{q}}. \end{array}$$

For each *q*, each estimated α(*q*) appears in the multifractal spectrum only when Shannon entropy of μ(*q,L*) scales with *L* according to the Hausdorff dimension *f* (*q*),where

(3) $$\begin{array}{rcl} f\left(\alpha \left(q\right)\right) & = & -\underset{N_{j}\to \infty }{lim}\frac{\overset{N_{j}}{\underset{i=1}{\sum }}\mu_{ij}\left(q,L_{j}\right)ln\left[\mu_{ij}\left(q,L_{j}\right)\right]}{lnN_{j}}\\ f\left(\alpha \left(q\right)\right) & = & \underset{L_{j}\to 0}{lim}\frac{\overset{N_{j}}{\underset{i=1}{\sum }}\mu_{ij}\left(q,L_{j}\right)ln\left[\mu_{ij}\left(q,L_{j}\right)\right]}{lnL_{j}} \end{array}$$

and where

(4) $$\begin{array}{rcl} \alpha \left(q\right) & = & -\underset{N_{j}\to \infty }{lim}\frac{\overset{N_{j}}{\underset{i=1}{\sum }}\mu_{ij}\left(q,L_{j}\right)ln\left[P_{ij}\left(L_{j}\right)\right]}{lnN_{j}}\\ \alpha \left(q\right) & = & \underset{L_{j}\to 0}{lim}\frac{\overset{N_{j}}{\underset{i=1}{\sum }}\mu_{ij}\left(q,L_{j}\right)ln\left[P_{ij}\left(L_{j}\right)\right]}{lnL_{j}} \end{array}$$

For −300 ≤ *q* ≤ 300, and including only linear relationships with correlation coefficient *r* > 0.995 for Equations 3, 4, the downward-opening curve [α(*q*)*,f* (*q*)] is the multifractal spectrum. α_*max*_-α_*min*_ is multifractal-spectrum width *w*_*MF*_ according to the CJ algorithm.

##### Calculating t_MF_ from comparison to iterated amplitude adjusted fourier-transform (IAAFT) surrogates

Fifty IAAFT surrogates [26] were produced for each original series, using 1,000 iterations of randomizing the phase spectrum from the Fourier transform, taking the inverse Fourier transform of the original series' amplitude spectrum with the randomized phase spectrum, and replacing the inverse-Fourier series with rank-matched values of the original series. We calculated *t*_*MF*_ as the difference $\left(w_{MF}-\left(\frac{1}{50}\right)\overset{50}{\underset{i=1}{\sum }}w_{Surr}\left(i\right)\right)$ divided by the standard error of *w*_*Surr*_. Hence, positive or negative *t*_*MF*_ indicated wider or narrower, respectively, spectra than surrogates.

#### Mouse-tracking analysis

E-Prime® software collected (x,y) mouse-tracking data at approximately 58 Hz. The R package “mousetrap” calculated x-position flips, area under the curve (AUC), and maximum displacement (MD) statistics from mouse-tracking trajectories (Kieslich et al., 2017).

##### Traditional geometric mouse-tracking measures

Each of the three classic mouse-tracking behaviors reflects deviations from the quickest, shortest, most linear mouse-cursor paths from the start position at the bottom middle of the screen to the top right or top left choices. That is, the mouse-tracking behaviors encode nonlinearity of mouse-tracking trajectory, but these three vary in what aspect of the nonlinearity they emphasize. Area under the curve (AUC) is the count of square-pixel units of screen space between the straightest linear path and the actual mouse-tracking movement enacted by the participant. Maximum displacement (MD) is the distance in pixels of the longest orthogonal line segment definable from the nonlinear trajectory and the shortest linear path. X-position flips is the count of reversals in horizontal direction, as though to suggest that each reversal reflects a mental vacillation between the two horizontally-spaced options of the forced-choice on the screen.

##### Information-theoretic entropy measures of mouse-tracking behaviors

We use Calcagnì et al.'s (2017) method of decomposing the mouse trajectories because Viswanathan and Kelty-Stephen (2018) had found that this decomposition was crucial for clarifying the effects of speech vs. tone contexts on the three classic metrics of AUC, MD, and x-position flips. Briefly, Calcagni et al.'s method computes two information-theoretic measures of entropy for each trial's mouse-tracking trajectory: one for slow movements ψ and one for fast movements ξ within the same trajectory. Viswanathan and Kelty-Stephen had found that speech-context effects on all mouse-tracking measures had all interacted with the slow entropy ψ. They interpreted this finding in light of their earlier finding that compensation for coarticulation became stronger for speech contexts, and they interpreted the significance of ψ interactions with speech context as indication of the fact that the use of speech context invoked longer-term processing, that is, processing that included perception both of the current target stimuli and the just-previous context stimulus. Despite finding this interesting interaction throughout all mouse-tracking behaviors, Viswanathan and Kelty-Stephen only found that x-position flips increased with ambiguity of the mid-continuum [ga]-to-[da], with a significant negative quadratic effect of continuum steps indicating a peak in the middle of the continuum.

Entropy measures can be sensitive to temporal correlations (Xiong et al., 2017), but mouse-tracking data was sampled at 58 Hz, and participant response occurred at an average of 650 ms. Hence, the mouse-cursor trajectories were roughly 30 to 40 samples long, generating series too short for judicious estimation of temporal correlations.

##### Entropy measures served as predictors of traditional geometric measures

X-position flips, AUC, and MD are all expected to increase with (Calcagnì et al., 2017) informational-entropy measures ψ and ξ quantify entropy for the entire trajectory and only for fast movements, respectively. Because all subsequent modeling includes both entropy measures in the same regression models, we refer to ψ as capturing slow entropy because any resulting effect of that measure will necessarily represent the contribution of entropy above and beyond ξ encoding fast-movement entropy.

#### Regression modeling

##### “GA” selection and response time (RT)

Linear mixed-effect modeling tested both hypotheses because RT and mousetrap measures varied continuously. Poisson mixed-effect modeling tested Hypothesis 1 because individual “ga” selections were dichotomous, and cumulative “ga” selections across 176 trials were better modeled as a count variable than as a continuous variable. Whereas logistic modeling uses logit links to model odds of a single dichotomous events, Poisson modeling uses log links to model the marginal probability of one more event. Poisson modeling allowed trial effects to explicitly model actual sequence of the dependent variable and allowed random-effects structure without compromising convergence in logistic models.

Throughout, mixed-effect modeling used random-effect intercepts per participant. Fixed-effect instances of linear and quadratic effects used orthogonal polynomials to eliminate correlation between them. In addition to predictor Step, other predictors were Context (i.e., Speech or Tone), and Precursor (Low or [aɹ] = 1, High or [al] = 2). All terms not including explicit interaction with Context refer to effects in the real-speech context case, that is, beyond the “simple replication” portion of Results, all modeling addresses all data including no-context cases. Context (Real-Speech) is the control case of the categorical variable Context, and it is customary for modeling to omit explicit listing of the control case (e.g., Bates et al., 2015).

##### Mouse-tracking measures (x-pos flips, MD, and AUC)

Linear mixed-effect modeling was used for mousetrap measures AUC and MD because these varied continuously. Poisson mixed-effect modeling was used for mousetrap measure x-position flip because x-position flips is a discrete and so count variable.

We attempted to model mouse-tracking behaviors as exhaustively as possible especially because we sought to attribute as much possible variability to non-multifractal trial-varying predictors. Hence, all models included a counterbalancing (CB) term dummy-coded as 0 or 1 depending on whether the “GA” choice appeared on the left or the right side of the screen. The placement of the response buttons should have as much to do with any pattern of the movement of the mouse cursor across the screen as any experimental stimulus features.

#### Availability of materials

All materials as well as the raw data supporting the conclusions of this manuscript will be made available by the authors, without undue reservation, to any qualified researcher.

## Results

### Effects of context multifractality on perceptual responses (proportion of “GA” selections) and response times (RT)

#### Logistic regression replicates the traditional CFC effects but finds no effect of context type

**Figure 6:**
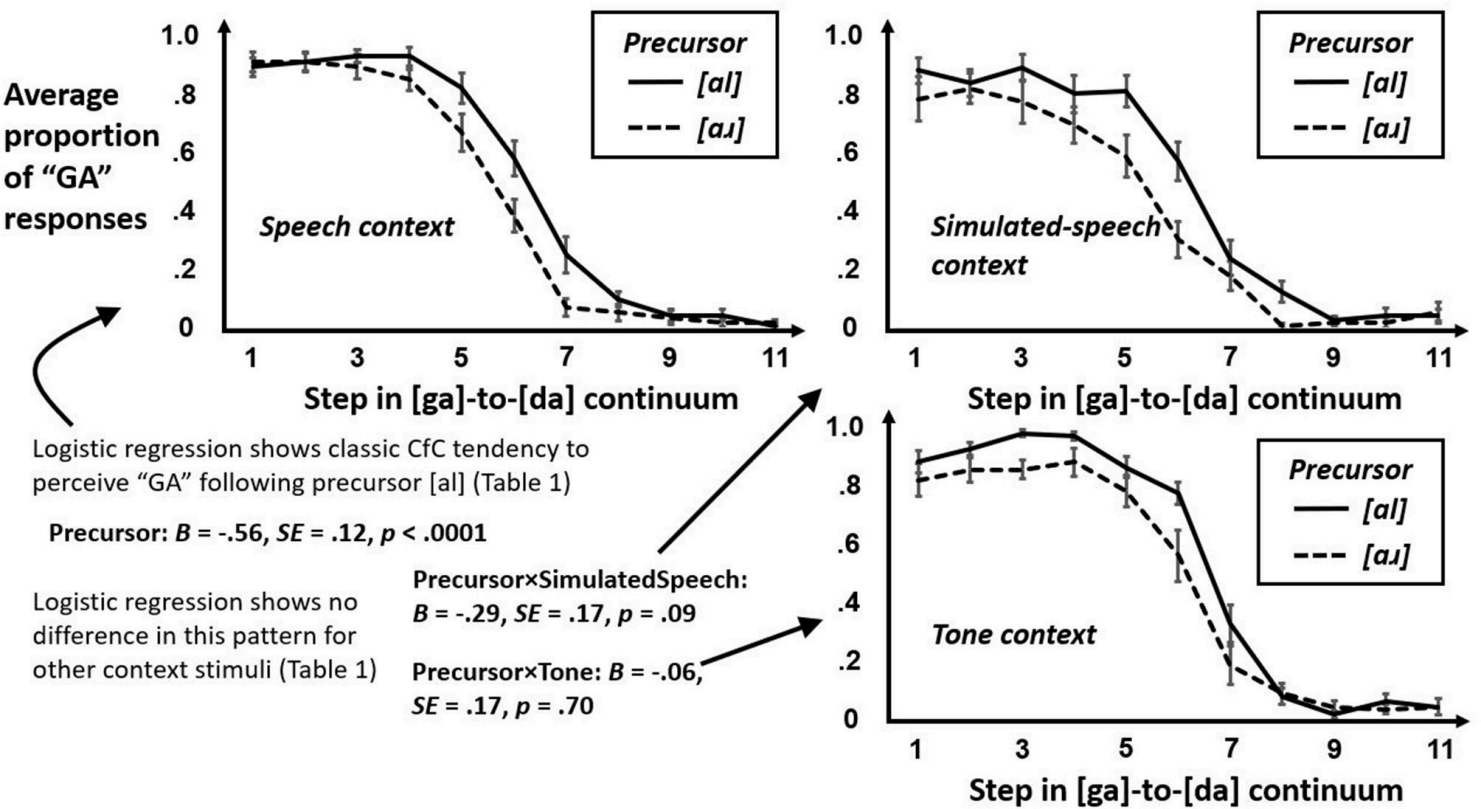
Average proportions of “GA” responses by precursor frequency [al] and [aɹ] plotted across Step in the [ga]-to-[da] continuum, for each of the three conditions in the study: real-speech context (top left panel), simulated-speech context (top right panel), and tone context (bottom right panel). Individual coefficients in the logistic regression in Supplementary Table 1 appear in bottom left panel to reinforce what the resemblance of these plots might already suggest, i.e., that the average proportions of “GA” response due to Precursor is not different following simulated-speech context or tone context than from following real-speech context. The approaching significance (p = 0.09) of the difference due to Precursor in the case of simulated-speech context only reflects an unstable tendency for the average proportions of “GA” response to drop off somewhat sooner for [aɹ] than for [al] from left to right along the Step continuum.

First, we sought to test whether participants heard “GA” more often following [al] than [aɹ]. This first step tested only whether [al] made hearing “GA” more likely on an average trial. A preliminary logistic regression (Supplementary Table 1) confirmed that “GA” responses become less likely with greater Step across the [ga]-to-[da] continuum and replicated the prior effect (e.g., Viswanathan et al., 2014; Viswanathan and Kelty-Stephen, 2018) that, on average, “GA” judgments were more likely following [al] and less likely than following [aɹ]. This model indicated no significant difference for tones or for simulated speech. Figure 6 shows the average proportion of “GA” response as a function of Step and plotted separately by precursor in each of three conditions. CfC effects are robust across different contexts. More subtle modeling will show that this portrayal in Figure 6 hides finer systematic differences across trial.

#### Comparable poisson modeling fails to replicate CFC effects

Next, we sought to test whether participants heard “GA” more often following [al] than [aɹ], but the difference here was that we wanted to test whether this effect was consistent across the entire experiment. Poisson modeling allowed previous research to document a cumulative effect of CfC (Viswanathan and Kelty-Stephen, 2018). Whereas logistic regression only tests for CfC effects to hold on average over all trials, Poisson modeling tests whether the effect of [al] precursors persists—across the entire experiment—in prompting a “GA” response more than otherwise. So, Poisson regression seeks to test whether the coarticulatory effects sustains similarly across all trials and does not merely hold on average, varying unsystematically across trials. However, perhaps strikingly, the Poisson regression modeling using the same predictors as the logistic regression in Supplementary Table 2 returned no effects for CfC and only a marginally significant tendency for simulated-speech contexts to discourage “GA” responses. Taken together, the significant effect of CfC in logistic modeling and the null effect for CfC in Poisson modeling means the following: the CfC effect is unstable and intermittent across time.

As shown in Supplementary Table 3, expanding the model to include block and trial effects improve model fit but fail to reveal any CfC effects and show no new differences in response due to simulated vs. natural speech.

#### Context multifractality's contributions to “GA” responses reveals CfC-related effects

The prior models cast doubts on whether participants heard “GA” more often following [al] than [aɹ]—that is, whether CfC was a really consistent, systematic effect across the entire experiment. Our next step in this modeling is to test whether the context multifractality was a crucial factor in whether participants heard “GA” or not.

CfC-related effects only appeared in Poisson modeling with the incorporation of predictors encoding context-stimuli multifractal estimates, i.e., *W*_*MF*_ (*B* = −0.34, *SE* = 0.17), *t*_*MF*_ (*B* = 0.06, *SE* = 0.03) and their interaction *W*_*MF*_ × *t*_*MF*_ (*B* = −0.39, *SE* = 0.18) were all significant at *p* < 0.05 (Table 2). What these results indicate is that likelihood of “GA” responses decreases following relatively more multifractal contexts but depends as well on the multifractality having nonlinear sources, i.e., on the *t*_*MF*_ being non-zero. The negative main effect of *t*_*MF*_ suggests a modest increase in likelihood of “GA” responses with more positive *t*_*MF*_, but the positive interaction suggests that the likelihood of “GA” decreases as *w*_*MF*_ and *t*_*MF*_ increase together. Table 1 details the model-predicted change in probability of “GA” response following all contexts of different multifractality. It is noteworthy that these model-predicted changes in probability change with [al] and [aɹ] but only does so because the different precursor contexts had different multifractal structure. This table reports only on the effects of multifractal measures, especially as there were no interactions of multifractal estimates with any other speech properties. Specifically, for both real-speech and simulated-speech contexts, the multifractality of the context stimuli predicted roughly 91% and 70% probability of “GA” response for [al] rather than following [aɹ]. Meanwhile, the multifractality of the tone-contexts predicted a similar decrease of probability over the lower and smaller range of 71% and 37%. Hence, despite the fact that Precursor × Context(Tone) has the opposite direction for real-speech and simulated-speech contexts, the multifractal effects on probability of “GA” response with different tone-contexts follows the same direction across precursors [al] and [aɹ] expected from real-speech and simulated speech. Hence, the Precursor × Context(Tone) effect stands in direct contrast to effects of multifractality from the context stimuli.

**Table 2:** All coefficients from Poisson regression predicting cumulative “GA” responses with multifractal estimates.

**Predictor**	***B***	***SE***	***p***
Intercept	8.19	3.19	<0.05
Context(Tone)	−6.26	3.17	<0.05
Context(SimulatedSpeech[SS])	−0.3022	0.15	<0.05
Linear(Step)	−0.0071	0.0018	<0.0001
Precursor	−1.24	0.70	0.07
Precursor × Context(Tone)	1.99	0.92	<0.05
Precursor × Context(SS)	−0.04	0.02	<0.05
**OVER-TIME EFFECTS: BLOCK NUMBER, TRIAL NUMBER WITHIN BLOCK, AND INTERACTIONS**			
Trial	0.03	0.0009	<0.0001
Block	0.31	0.0027	<0.0001
Block × Trial	−0.0035	0.0001	<0.0001
Block × Context(Tone)	−0.0030	0.0021	0.16
Block × Context(SS)	0.02	0.0023	<0.0001
Block × Linear(Step)	0.0010	0.0003	<0.001
**MULTIFRACTAL ESTIMATES: MULTIFRACTAL SPECTRUM WIDTH, T-STATISTIC INDICATING NONLINEARITY, AND INTERACTION**			
*W_*MF*_*	−0.34	0.17	<0.05
*t_*MF*_*	0.06	0.03	<0.05
*W_*MF*_*×*t_*MF*_*	−0.39	0.18	<0.05

**Figure 7:**
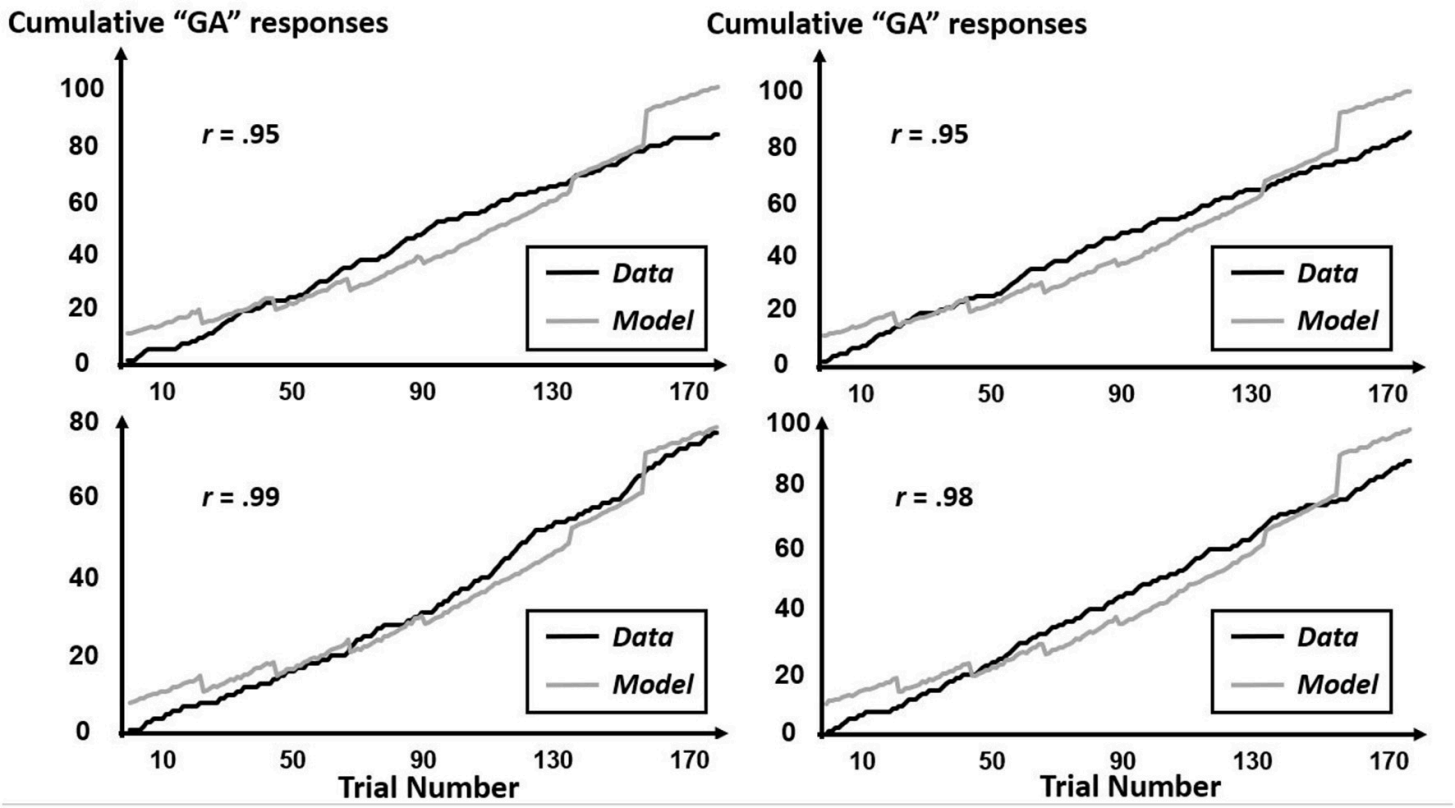
Cumulative “GA” judgments and corresponding model predictions for four individual participants in the real-speech context. To depict the full range of regression-model performance in the real-speech condition, we include individual-participant plots from the two participants for whose data the model fit worst (top two panels) as well as from the two participants for whose data the model fit best (bottom two panels).

**Figure 8:**
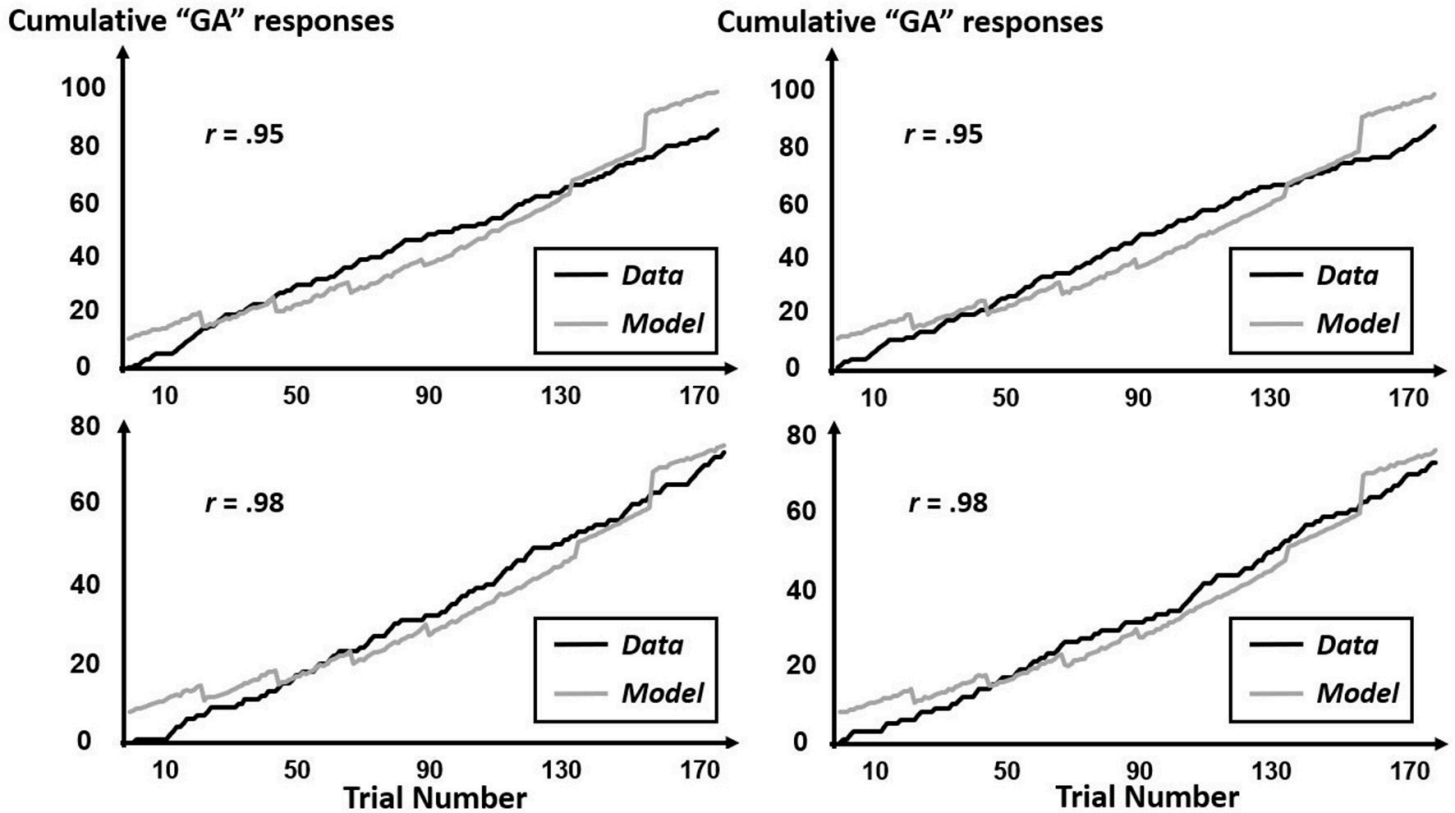
Cumulative “GA” judgments and corresponding model predictions for four individual participants in the tone context. To depict the full range of regression-model performance in the tone condition, we include individual-participant plots from the two participants for whose data the model fit worst (top two panels) as well as from the two participants for whose data the model fit best (bottom two panels).

**Figure 9:**
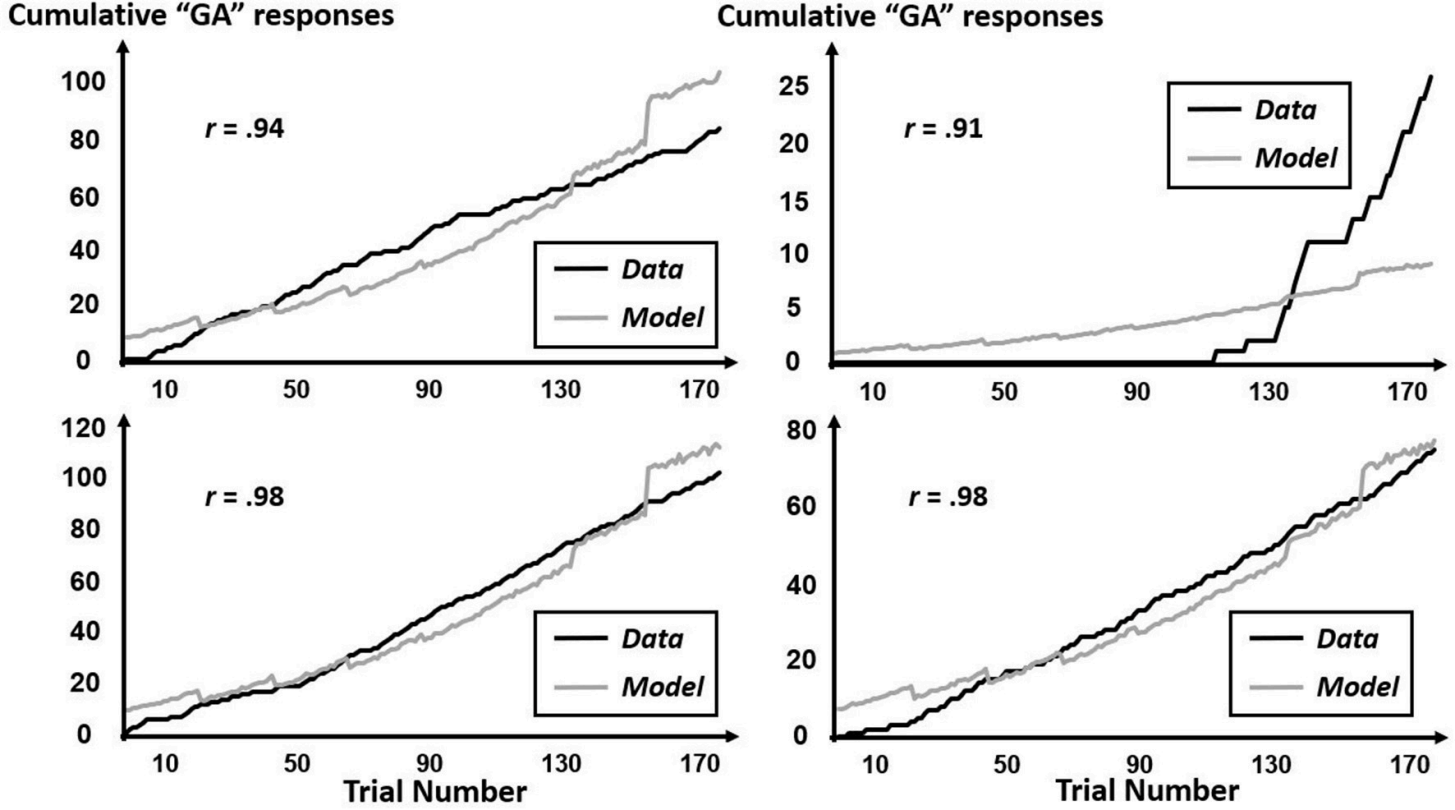
Cumulative “GA” judgments and corresponding model predictions for four individual participants in the simulated-speech context. To depict the full range of regression-model performance in simulated-speech condition, we include individual-participant plots from the two participants for whose data the model fit worst (top two panels) as well as from the two participants for whose data the model fit best (bottom two panels).

The CfC-related effects revealed by this multifractal elaboration only appeared significantly for the interactions of precursor with tone context (*B* = 1.99, *SE* = 0.92) and of precursor with simulated-speech context (*B* = −0.04, *SE* = 0.02) with both *p*s < 0.05, and real-speech context only contributed to a marginally significant CfC effect (*B* = −1.24, *SE* = 0.70, *p* = 0.07). The positive interaction of precursor with tone contexts indicates that “GA” responses were increasingly more likely following tones approximating the F3 of [aɹ]. Meanwhile, the negative interaction with precursor and simulated-speech context aligned more closely with the prior understanding of “GA” responses being more likely following [al] rather than [aɹ] (Table 2). The negative interaction of precursor with real-speech context for Poisson modeling is analogous to the negative significant effect in the logistic regression, with the difference in significance perhaps indicating that the CfC effect of real-speech contexts does not persist throughout the entire experiment but only holds on average as indicated by the logistic regression. The absence of any Block interactions of precursor real-speech contexts suggest that there is no systematic growth or decrease of this CfC effect and that different participants exhibit different sequences of responses indicating CfC. Figures 7–9 display individual-participant model predictions in the real-speech context, tones context and simulated-speech context, respectively, for four participants in each condition.

Alternative Poisson modeling omitting the simulated-speech context condition did not find significant effects for tone or for real-speech contexts. Because the tone and real-speech contexts did not vary within their corresponding level of Precursor, differences in multifractal estimates were not appreciably different from differences in Precursor, and so unsurprisingly, adding multifractal estimates to Poisson models omitting simulated-speech context were no more effective at revealing significant effects for only tone and real-speech contexts than earlier Poisson models.

#### Response times (RT) responded to wider set of predictors than did likelihood of cumulative “GA” response

Having modeled whether participants heard “GA” or not, we then turned to modeling how much time participants took to register their perceptual response as a mouse-click on a corresponding icon on the screen. Because Poisson modeling including multifractal measures was successful for modeling whether participants heard “GA,” we thought that Poisson modeling including multifractal measures would also offer a successful way to model the time participants took to register this response.

Table 3 reports all of the significant contributions to RT. As noted in the Method section we used Poisson regression because it is a method for addressing non-negative count variables and it is applicable to continuous variables. Furthermore, whereas a traditional approach to RT is to use linear regression on logarithmically scaled RT, Poisson regressions make the logarithmic transformation implicit in its log-linking. With few exceptions noted below, all effects were significant at *p* < 0.0001, and all effects not included either did not make significant improvements to model fit or led model convergence to fail.

**Table 3:** All coefficients from Poisson regression predicting response times (RTs).

**Predictor**	***B***	***SE***	***p***
Intercept	−83.07	0.24	<0.0001
Context(Tone)	90.65	0.26	<0.0001
Context(SimulatedSpeech[SS])	−0.34	0.14	<0.05
Linear(Step)	7.98	0.15	<0.0001
Quadratic(Step)	−2.40	1.48	<0.0001
Precursor	−0.35	0.07	<0.0001
Precursor × Context(Tone)	−35.86	0.21	<0.0001
Precursor × Context(SS)	0.14	0.0043	<0.0001
Linear(Step) × Context(Tone)	–3.91	0.10	<0.0001
Quadratic(Step) × Context(Tone)	–6.05	0.09	<0.0001
Linear(Step) × Context(SS)	–3.66	0.09	<0.0001
Quadratic(Step) × Context(SS)	–2.30	0.09	<0.0001
**OVER-TIME EFFECTS: BLOCK NUMBER, TRIAL NUMBER WITHIN BLOCK, AND INTERACTIONS**			
Trial	−0.04	0.0001	<0.0001
Block × Trial	0.0062	0.0001	<0.0001
Block	0.15	0.0018	<0.0001
Block × Context(Tone)	−0.04	0.0014	<0 0.0001
Block × Context(SS)	−0.02	0.0004	<0.0001
Block × Linear(Step)	−1.30	0.02	<0.0001
Block × Quadratic(Step)	0.51	0.02	< 0.0001
**MULTIFRACTAL ESTIMATES: MULTIFRACTAL SPECTRUM WIDTH, T-STATISTIC INDICATING NONLINEARITY, AND INTERACTION**			
*W_*MF*_*	357.40	1.02	<0.0001
*t_*MF*_*	−0.6.96	0.05	<0.0001
*W_*MF*_*×*t_*MF*_*	36.74	0.25	<0.0001
*W_*MF*_*× Precursor	260.87	0.75	<0.0001
*t_*MF*_*× Precursor	1.56	0.01	<0.0001
*W_*MF*_*× Block	0.02	0.01	<0.05
*t_*MF*_*× Block	0.0017	0.0001	<0.0001

##### Effects of context and CfC-related terms

Compared to RT for real-speech context, RT was higher following tone contexts (*B* = 90.65, *SE* = 0.26) and lower following simulated-speech contexts (*B* = −0.34, *SE* = 0.14, *p* < 0.05). As for precursor effects, [aɹ] prompted quicker responses than [al] (*B* = −0.35, *SE* = 0.07) especially for tone contexts (*B* = −35.86, *SE* = 0.26), but simulated-speech contexts sped responses least (*B* = 0.14, *SE* = 0.0003). The significant negative quadratic effect of Step (*B* = −2.40, *SE* = 1.48) indicated significantly slower response for the middle steps of the [ga]-to-[da] continuum which grew slower in the case of tone and simulated-speech contexts (*B*s = −6.05 and −2.30, *SE*s = 0.09, respectively). Difference in these negative coefficients indicated that response slowness for middle-step [ga]-to-[da] tokens was greatest especially following tone contexts. These effects held above and beyond the lower-order linear effects of Step and its interactions with tone and simulated-speech context (*B*s = 7.98, −3.91, and −3.66; *SE*s = 0.15, 0.10, and 0.09, respectively) but became progressively faster with subsequent Blocks (*B* = 0.51, *SE* = 0.02).

##### Block interactions with trial, context, and step

RT increased across blocks (*B* = 0.15, *SE* = 0.0018), decreased across trials within block (*B* = −0.04, *SE* = 0.0001), but this change with trials became shallower in later blocks and actually increased in Blocks 7 and increased faster in Block 8 (*B* = 0.0062, *SE* = 0.0001). RT increased less over blocks in both tone and simulated-speech conditions (*B*s = −0.04 and −0.02, *SE*s = 0.0014 and 0.0004, respectively). The above-mentioned negative relationship between RT and steps in the [ga]-to-[da] continuum dwindled on two counts: first, the linear effect of Step becoming weaker with blocks (*B* = −1.30, *SE* = 0.02) until reversing and becoming positive in Block 7 and growing in Block 8; and second, with the negative quadratic effect of Step going from negative to positive in Block 5 and growing larger in later blocks (*B* = 0.51, *SE* = 0.02). The absence of Block interactions with Step × Context indicate that responses remained slower in the middle [ga]-to-[da] steps for tone and simulated-speech contexts, but the eventually negative-linear, positive-quadratic profile of RT with Step following real-speech contexts entails gradually quicker responses for all but the strongest [ga] candidates in the [ga]-to-[da] continuum.

##### Multifractal effects on RT

RT increased with greater multifractal spectrum width *W*_*MF*_ (*B* = 35.74, *SE* = 1.02). Non-zero *t*-statistics indicating evidence of nonlinear sources of multifractality accentuated this *W*_*MF*_ × *t*_*MF*_ (*B* = 36.74, *SE* = 0.25). Whereas the model of “GA” responses showed no significant interactions of multifractal estimates with task parameters, the model of RT exhibited effects of *W*_*MF*_ × Precursor (*B* = 260.87, *SE* = 0.75) and *t*_*MF*_ × Precursor (*B* = 1.56, *SE* = 0.01). Table 1 depicts how the model-predicted probability of an additional millisecond of RT per trial changed with the multifractal properties of the context stimuli. Multifractal effects predicted greater probability (1,637%) of single-millisecond increases in RT for real-speech contexts and lesser probabilities (1,483 and 1,490%) of single-millisecond increase in RT for tone and simulated-speech contexts, with miniscule differences across precursor in real-speech context, slightly greater range across precursors with tone context and among 8 variants of the [aɹ] precursor, and greatest range among the 8 variants of the [al] precursor.

##### Multifractal interactions with block

What Table 1 does not show is the additional results that RT increased with block's interactions with *W*_*MF*_ (*B* = 0.02, *SE* = 0.01, *p* < 0.05) and with *t*_*MF*_ (*B* = 0.0017, *SE* = 0.0001). Hence, with further blocks in the experiment, RT increased more so with multifractal-spectrum W_MF_ and to a lesser degree with *t*_*MF*_.

#### Discussion of perceptual response and RT, and hypotheses for second section

We tested two predictions: first, simulated speech will prompt weaker differences in “GA” selection between [al] and [aɹ] as well as faster responses (Hypothesis 1), and second, judgment of target speech syllables following a context will depend on the multifractal unevenness across time in context sounds, the degree of nonlinearity contributing to that multifractal unevenness, and the interaction of these two components, and this difference should appear both in the accumulation of “GA” judgments across the experiments as well as the accumulation of response time in making these judgments (Hypothesis 2). Results supported all predictions. It is worth emphasizing that our support of Hypothesis 1 is a replication of the findings by Viswanathan and Kelty-Stephen (2018). It is equally important to stress that the support for Hypothesis 2 was crucial for supporting this replication of Hypothesis 1. Simply put, there were no effects of CfC until modeling was cumulative rather than on-average (i.e., Poisson rather than logistic, respectively), and there were not effects of CfC in Poisson modeling until we incorporated multifractal estimates w_MF_ and t_MF_. The effects of multifractality on compensation for coarticulation appear to support the replicable facts of compensation for coarticulation.

Perhaps the most striking result in the foregoing was the initial failure to replicate the coarticulation compensation as noted by Viswanathan and Kelty-Stephen (2018). Viswanathan and Kelty-Stephen found both compensation for coarticulation effects in logistic regressions testing average proportion of “GA” response and interactions of time with compensation for coarticulation in Poisson modeling of cumulative “GA” responses. The present work found compensation for coarticulation only in the logistic regression and found no difference by context in the average proportions of “GA responses.” The present Poisson model found no effect for compensation for coarticulation, even after the inclusion of time effects to interact with the compensation for coarticulation.

Crucially, it was only the inclusion of multifractal estimates *w*_*MF*_, t_MF_, and their interaction that revealed effects of compensation for coarticulation in the Poisson model for “GA” responses. The prior failure of the Poisson modeling suggests that the compensation for coarticulation was uneven over time, but it is intriguing that including multifractal estimates manages to control for this and reveal the known significant effects related to compensation for coarticulation. One possible explanation for this pattern of results is that multifractal estimates indicate unevenness with time, and the unevenness with time found in context stimuli might serve to produce the unevenness with time found in the “GA” responses. Said another way, the failure to replicate the simpler, multifractal-free effects showing that coarticulation for coarticulation need not be troubling because the nonlinear interactions across time scale in the measured behaviors—both in speech context and in participants' “GA” responses—are classically statistical mechanisms known to produce intermittent, nonstationary, and irregular results over average (Wallot and Kelty-Stephen, 2017).

Strange though it may sound, multifractality may be the geometry that defines the landscape of events shaping our perceptual experience (Kelty-Stephen and Dixon, 2014). Whether or not each of those events has sufficient strength to activate individual neurons and whether or not all details of this multifractal geometry reaches our current awareness, it may be that the multifractal structure shapes the longer-range perceptual use of available information—beyond the time scale of individual neuron firings and into the time scales of the following, coarticulated phonemes. The picture of speech perception coming into focus is that of a multifractal patterning that arises from the details of the movement system producing speech. The next section of the present work raises the possibility that, on the way to the participant's decisions about speech-perception, it may be the listener's movement system that acts as a substrate to absorb the multifractality in context sounds and carry them forward to the mouse-clicked response. Hypothesis 3 will be that mouse-cursor movements by the participant will reflect the interactions of multifractal measures and Context with linear effects of Step, quadratic effects of Step, Block, Trial, Precursor, as well as Calcagnì et al.'s (2017) entropy measures. Hypothesis 4 will be, as in Viswanathan and Kelty-Stephen (2018) a negative main effect of quadratic effects of Step would appear in the measure of x-position flips.

### Effects of context multifractality on mouse-cursor trajectories on the way to clicked response

In this section, we review modeling of the mouse-cursor trajectory data as participants move the mouse-cursor to the appropriate icon on the computer screen to register their response. We already found that context multifractality influenced whether participants heard “GA” and how much time participants took to register this response. The present modeling is important to the present considerations because it would test our prediction that context multifractality would also influence the movement system of the listening participants, particularly as they used their perception of speech to make a task response.

#### Maximum displacement (MD) and area under the curve (AUC)

Our model for MD was specified by the following family of highest-order interactions:

CB × ψ × ξ × Linear(Step) × Precursor × Context × Block × Trial
CB × ψ × ξ × Quadratic(Step) × Precursor × Context × Block × Trial
ψ × ξ × W_MF_×Linear(Step) × Precursor × Block
ψ × ξ × W_MF_×Quadratic(Step) × Precursor × Block
ψ × ξ × t_MF_×Precursor × Block.

The model included these higher-order interactions named above, as well as all lower-order interactions and main effects composing these higher-order interactions. No other effects improved model fit. Supplementary Table 4 lists their significant coefficients (*p* < 0.05), organized by whether they implicate W_MF_ or t_MF_, the interaction of ψ and ξ without any multifractal effects, and the interaction of CB with all non-multifractal effects.

Our model for AUC was specified by the following family of highest-order interactions:

CB × ψ × ξ × Precursor × Context × Block
W_MF_×t_MF_×ψ × ξ × Linear(Step) × Precursor × Context × Block × Trial
W_MF_×t_MF_×ψ × ξ × Quadratic(Step) × Precursor × Context × Block × Trial

The model included these higher-order interactions named above, as well as all lower-order interactions and main effects composing these higher-order interactions. No other effects improved model fit. We were able to fit much higher-order interactions that significantly improved model fit here in the model for AUC than in the foregoing model for MD. One explanation for this difference is AUC has greater capacity to vary than MD: MD varies proportionally to the number of pixels in one direction, and as an area measure, AUC varies as pixels-squared. So, AUC may simply be a dependent measure that manifests more clearly the expression of a greater number of independent factors. Beyond the capacity for a larger stable model, an interesting feature of this model is that the significant interaction of W_MF_ and t_MF_ with one another as well as with all other factors besides counterbalancing (CB). Supplementary Table 5 lists their significant coefficients (*p* < 0.05).

The results of this model indicated that MD and AUC of mouse-tracking behavior depended on the multifractality of the context stimuli, on type and frequency of the context stimuli, on the step in the [ga]-to-[da] continuum, and on the different speed of movements within a single trial's mouse-tracking behavior (i.e., the entropy-based parsing of the movement series). A skeptical view might consider it plausible that this sensitivity of MD and AUC to multifractality might fall within incidental variability of MD and AUC due to the direction of movement across the screen. For this reason, Supplementary Tables 4, 5 include all CB-related interactions to confirm that screen position did have a significant impact on mouse-tracking movements but to emphasize that multifractal effects on mouse-tracking movements did not depend on screen position. However, we omit explicit discussion of them except to note here that effects of multifractality did not depend on any factors incidental to counterbalancing of screen position.

##### MD showed dual-entropy interactions with all non-counterbalancing factors and single-entropy interactions with specific types of multifractal estimates

Far from being straightforwardly smaller with more practice and perhaps with more confidence on later trials, the observed MD appears to depend strongly on the different contexts and the different precursor frequencies, and it appears to do so differently in the slower and faster components (i.e., ψ and ξ, respectively) of the mouse-tracking behaviors. Supplementary Figures 1, 2 depict the specific effects of interactions involving multifractal estimates W_MF_ and t_MF_, respectively, as well as with entropy measures, Block, Trial, and Step predictors. Important to note, Supplementary Figures 1, 2 show predicted MD for the W_MF_-based effects in the specific case of Step = 5 in the middle of the 0-to-10 11-step continuum.

The prevalent case of non-CB effects was the interaction of both entropies together ψ × ξ with other factors. The relatively rare exceptions to ψ × ξ supporting the significant interaction effects were five interaction terms only featuring one entropy: ψ × W_MF_×Precursor × Block, ξ × t_MF_, ξ × t_MF_×Block, ξ × t_MF_×Precursor, and ξ × t_MF_×Precursor × Block. Prior work had found significant interactions of ψ only with speech context rather than tone context. The present work finds now that interactions of entropy-based measures ψ-alone were significant for W_MF_-related effects only and ξ-alone were significant for t_MF_-related effects only.

##### MD showed linear effects of step for all non-counterbalancing effects and quadratic effects of step for counterbalancing effects

The traditional interpretation of MD as indicating uncertainty or increasing processing load would suggest that MD should increase for middle steps of the [ga]-to-[da] continuum, but the absence of a quadratic effect indicates no significant difference for the middle steps. The linear effect of Step only shows changes in MD with increasing or with decreasing Step. The interaction implicating Linear(Step) with W_MF_ (i.e., ψ × ξ × W_MF_×Linear(Step) × Precursor × Block; *B* = −8.87 × 10^8^; *SE* = 3.46 × 10^8^) is negative: MD decreased with increased Step as well as with increases in either entropy and in *W*_*MF*_. This interaction was stronger for [aɹ] than for [al] and, in either case, with increasing block number.

The next set of interactions involving Linear(Step) were those interactions including neither multifractality nor CB effects. Two effects in this set indicated a MD increase, i.e., ψ × ξ × Linear(Step) × Precursor × Block and ψ × ξ × Linear(Step) × Precursor × Block × Trial. Another three effects in this set indicated decreases in MD for similar interactions specific to the tone-context condition, i.e., ψ × Linear(Step) × Context(Tone) × Block × Trial, ψ × ξ × Linear(Step) × Precursor × Context(Tone) × Block, and ψ × ξ × Linear(Step) × Precursor × Context(Tone) × Block × Trial.

##### Major distinctions among effects on AUC

As with MD, far from being straightforwardly smaller with more practice and perhaps with more confidence on later trials, the observed AUC appears to depend strongly on the different contexts and the different precursor frequencies, and it appears to do so differently in the slower and faster components (i.e., ψ and ξ, respectively) of the mouse-tracking behaviors. Rather than attempt to detail the extensive significant individual findings in text, we make relatively few distinctions to organize the AUC findings. Supplementary Figure 3 to depict the specific effects of interactions involving multifractal estimates W_MF_ and t_MF_ with each other as well as with entropy measures, Block, Trial, and Step predictors. Important to note, Supplementary Figure 3 shows predicted AUC for these effects in the specific case of Step = 5 in the middle of the 0-to-10 11-step continuum.

###### AUC showed CB interactions with context but not step vs. multifractal interactions with step but not context.

One major distinction we can draw amongst these effects is that, whereas W_MF_, t_MF_, and W_MF_×t_MF_ interact with the quadratic term of Step but not with Context, CB interactions include interactions with Context but not with the Step terms.

###### AUC showed analogous multifractal-dependent interactions involving W_*MF*_ only or t_*MF*_ had opposite signs.

A second distinction is different subsets of the effects mirrored one another's directionality. There were many effects involving either W_MF_ or t_MF_ but not both, and some of these interactions were identical except for including an interaction with W_MF_ for an interaction with t_MF_. These analogous interactions showed mutually opposite effects. For instance, Supplementary Table 5 lists a significant interaction ψ × ξ × W_MF_ (*B* = 7.60 × 10^6^; *SE* = 3.64 × 10^6^) but also a significant interaction ψ × ξ × t_MF_ (*B* = −5.40 × 10^4^; *SE* = 2.58 × 10^4^), and coefficients for these similarly built terms have opposite signs. The opposite signs here reflect independent and separable contributions of correlated factors W_MF_ and t_MF_.

W_MF_ × t_MF_ interactions had opposite signs from analogous interactions without multifractal terminology. For instance, Supplementary Table 5 lists a significant interaction ψ × ξ × W_MF_ × t_MF_ (*B* = 4.87 × 10^5^; *SE* = 2.40 × 10^5^) as well as a significant interaction ψ × ξ (*B* = −8.39 × 10^5^; *SE* = 3.87 × 10^5^).

#### X-position flips

Our model for AUC was specified by the following family of highest-order interactions and main effects: Precursor × Context, ψ × W_MF_, ξ × W_MF_, Precursor × W_MF_, Block × Trial, Linear(Step), Quadratic(Step) and CB.

The model included these higher-order interactions named above, as well as all lower-order interactions and main effects composing these higher-order interactions. No other effects improved model fit. Whereas AUC afforded much more variability than MD within which to fit additional significant predictors, x-position flips proved much less forgiving than MD in terms of affording the variability to warrant many significant predictors. Supplementary Table 6 lists all coefficients (*p* < 0.05).

This much sparser model allows us to detail the entirety of the model's coefficients. First, the interpretation of x-position flips as a sign of uncertainty finds some confirmation in that they are more frequently found in the slower component of mouse-tracking movements (ψ; *B* = 1.38, *SE* = 0.08, *p* < 0.0001) and much less likely in the faster component of mouse-tracking movements (ξ; *B* = −0.64, *SE* = 0.09, *p* < 0.0001). We fit the lower-order linear effect of Step on the [ga]-to-[da] continuum (*B* = −8.92, *SE* = 1.43, *p* < 0.0001) as a means to get a proper estimate for the quadratic effect of Step (*B* = −2.85, *SE* = 1.40, *p* < 0.05). The negative linear effect entails that x-position flips are much more likely for [ga] than for [da] sounds, but the negative quadratic effect of Step confirms that x-position flips do increase the most for the middle steps of the [ga]-to-[da] continuum, i.e., for the most ambiguous stimuli. As for effects of context, x-position flips are significantly less frequent following a tone context (*B* = −43.86, *SE* = 2.87, *p* < 0.0001) than during either real-speech or simulated-speech contexts, neither of which latter two different from one another. X-position flips were more likely following [al] (Precursor = 1) and less likely following [aɹ] (Precursor = 2; Precursor, *B* = −18.81, *SE* = 1.60, *p* < 0.0001) for real-speech and simulated-speech contexts. The tone context showed none of this dependence of x-position flips on precursor (*B* = 18.20, *SE* = 1.30, *p* < 0.0001). X-position flips are less likely following context stimuli with greater multifractality W_MF_ (*B* = −287.80, *SE* = 15.50, *p* < 0.0001), particularly in the slower components of mouse-tracking behavior (*B* = −2.77, *SE* = 0.71, *p* < 0.001). This finding might suggest a direct relationship between multifractality of the context stimuli and the confidence of the decision-making, but it is important to note that the multifractality may both increase x-position flips in the faster components of mouse tracking behavior (*B* = 2.33, *SE* = 0.80, *p* < 0.01) and may have a weaker effect in diminishing x-position flips following context stimuli associated with [aɹ] (Precursor = 2) rather than with [al] (Precursor = 1; *B* = 103.10, *SE* = 21.06, *p* < 0.0001). No effects of t_MF_ significantly improved model fit. Supplementary Figure 4 depicts the average differences in x-position flips for different entropy and for different precursor.

Across the experiment, x-position flips decrease with increasing blocks (*B* = −0.11, *SE* = 0.01, *p* < 0.0001) revealing either growing tedium or growing resoluteness regarding how participants choose to respond to each item. There is as well a decrease in x-position flips with increasing trial number within blocks (*B* = −0.03, *SE* = 0.0051, *p* < 0.0001) that diminishes across blocks (*B* = 0.0042, *SE* = 0.0011, *p* < 0.001). We left in the nonsignificant main effect of CB to show that counterbalancing of screen position does not change x-position flips as a main effect, and no additional interaction of multifractal effects with CB improved model fit either.

#### Discussion

We hypothesized that multifractal measures contributed to all interactions of Context with linear effects of Step, quadratic effects of Step, Block, Trial, Precursor, as well as (Calcagnì et al., 2017) entropy measures (Hypothesis 3), and we also hypothesized that the only negative main effect of quadratic effects of Step would appear in the measure of x-position flips (Hypothesis 4). Results supported both hypotheses. None of these effects are artifactually due to counterbalancing of the “DA” and “GA” locations on the screen because all modeling incorporated this effect with its CB predictor.

##### Hypothesis 3

Both MD and AUC showed a vast set of significant effect of interactions involving multifractal measures W_MF_ and t_MF_ with Context, linear effects of Step, quadratic effects of Step, Block, Trial, Precursor, as well as Calcagnì et al.'s (2017) entropy measures. An important distinction among these two measures is that the statistical model for AUC exhibited a dependence on the interaction of W_MF_ with t_MF_, which two-way interaction term participated in many higher-order interactions with the other predictors. On the other hand, the statistical model for MD only supports interactions with W_MF_ and also, independently from those interactions, interactions with t_MF_. These independent sets of interactions show a similar pattern of results indicating that these different facets of multifractal structure pick up on similar groupings of predictor variables. It may also be that availability of the W_MF_×t_MF_ interactions in the AUC case may reflect the fact that the AUC definition in terms of pixels-squared affords greater margins predictable variability than the MD defined in terms of pixels. That is to say, the higher dimensional definition of AUC than of MD may explain some of the asymmetry between regression-modeling results for each of the measures.

##### Hypothesis 4

The model of x-position flips shows an interesting case in which the multifractal structure in terms of W_MF_ completely replaces the role of Context in any interaction with (Calcagnì et al., 2017) entropy measures that Viswanathan and Kelty-Stephen (2018) had shown. Generally, the effect of multifractality seems to be to reduce the number of x-position flips particularly in the slower components of mouse-tracking movements. Also, it seems to reduce the number of x-position flips more so in the case of [al] than for [aɹ].

### General discussion

As noted above, the main idea of this research is to investigate whether the multifractality (w_MF_) and the nonlinearity (t_MF_) in speech-production is an important support for speech perception using speech contexts. This main idea promises to elaborate an explanation for why speech contexts may be better, in the long-run, for supporting compensation for coarticulation. We estimated the multifractal signatures specific to the movement system producing speech as well as estimating multifractal signatures specific to speech resynthesized to destroy sequence while preserving phoneme-category membership. We tested whether multifractal signatures predicted perceptual response (i.e., which phoneme participants chose) but also whether those multifractal signatures predicted the participants' mouse-cursor trajectories as they moved to click the icon corresponding to their choice. All tests suggested that multifractal estimates of context stimuli did in fact influence the perceptual response and the mouse-cursor trajectory as participants moved to register those responses.

The current model of compensation for coarticulation in general-auditory accounts has been that the formant offsets of vowel-consonant contexts strike a contrast with formant onsets of subsequent consonant-vowel. What we have accomplished is to show that compensation for coarticulation shows subtle but significant disturbance when resynthesizing speech context sounds reorders the vowel in the context but leaves the subsequent formant offsets and onsets implicated in contrast unchanged. Hence, whatever spectral contrast might contribute to compensation for coarticulation, these effects rest within a deeper, longer-range context of multifractal structure of context sounds.

No matter their subtlety, the multifractal effects may be key for finding any significant interaction of context with formant offset (i.e., precursor) over trials in an experimental study. The logistic regression on “GA” responses only shows an effect of Precursor indicating the role of formant offset without any further interaction of formant offset with context. This finding would seem to support a general-auditory account of compensation for coarticulation by finding a change in “GA” response with spectral contrast and no change in “GA” response with context. However, a comparable Poisson regression for modeling this effect across trials in the experiment shows no significant effect of formant offsets (Precursor) but a moderate negative effect of simulated-speech contexts. The difference in results is evidence that the compensation for coarticulation holds on average, but it is not a stable result across trials.

One key for decoding and unlocking the meaning from this instability of spectral-contrast across each and every trial is multifractal analysis. The instability of the effect of formant-offsets across trials can be understood as soon as the model incorporates multifractal estimates W_MF_, t_MF_, and W_MF_×t_MF_. That is, the instability of the context sounds across time (W_MF_), the instability of context sounds specifically due to nonlinearity (t_MF_), and their relationship—all three of these components need only to appear in the Poisson regression model, and when they do, then the significant effects for context and precursor emerge in just the pattern that we typically expect from comparable Poisson modeling of previous results (e.g., Viswanathan and Kelty-Stephen, 2018). It is important to note that the contribution of multifractal effects acts above and beyond already included effects of Block and Trial and their interactions with the preexisting predictors. It is no less important to note that these multifractal effects do the same job without having added in those Block- and Trial-related effects. Hence, the multifractal effects appear to be important underlying factors for understanding compensation for coarticulation in speech perception.

### Two kinds of context: the just-previous context of general auditory theories and the (nonlinear) interactions-across-time-scales from gestural theories of language

Context effects prompt speech-perception researchers to operationalize context in two very different ways. Researchers from a “general auditory process” perspective eschew any distinctions between speech sounds and other auditory stimuli (e.g., Laing et al., 2012), and they situate the “context” very cleanly completely in the recent, just-previous sounds (Stilp and Assgari, 2018). Hence, they propose a model of speech perception that should be like any other short-memory Markov sequence of acoustic processing in which nothing but extremely recent spectral properties carries any effects on current perceptions (e.g., Chambers et al., 2017). The “gestural” perspective on speech perception emphasizes that what people perceive in speech are articulatory gestures, i.e., not the “gestures” including hand movements and head tilts but the rather the patterns of movement that generate speech sounds. For gestural theories, compensation for coarticulation reflects speech perception's capacity to bypass low-dimensional acoustic variability (i.e., in frequency means or intensity patterns) and to find invariant structure in gestures (Fowler, 2006). These gestures set up a context that shapes the perception of a target phoneme, but because gestures are themselves context-sensitive, gestural theories encompass a broad set of structures unfolding and interacting with one another across a variety of time scales, e.g., the entire phrase in which a phoneme occurs (Tilsen, 2009), the surrounding discourse (Skipper et al., 2017), the facial movements implicated in articulation (Massapollo et al., 2018), the social setting and concurrent multimodal sensory information (Levinson and Holler, 2014), the language itself (Tobin et al., 2017). Compared with the extremely stringent just-previous-sound definition of context from general-auditory theorists, the gestural-theoretic context risks seeming sprawling and perhaps unfalsifiably vast.

Nonetheless, the narrowness of the general-auditory theoretic “context” severely ends up conflicting with plain evidence generic to all acoustic structure—leading to falsifiability challenges of its own. For instance, a variety of speech sounds (notably fricatives and stop consonants) involve the generation of turbulent fluid flow (Stevens, 1971; Mitra et al., 2017), suggesting that the acoustics of speech contain long-range correlations beyond short-memory processes. Making matters more challenging for a theory of speech perception drawing heavily on brief spectral properties, turbulent air flow from the vocal tract during a pause can exhibit spectral properties similar to the previously voiced phoneme. That is to say, turbulence does not simply entail long-range structure during voicing, but turbulence may actually force general auditory theorists into a false positive of registering a phoneme that is no longer voiced (Hlavnicka et al., 2017). Hence, speech actually embodies the capacity for turbulent fluid flow to fossilize past structures, freezing them (or even reconstituting them) after the driving forces originally producing them have vanished—which capacity is generic to fluid flows, acoustic or otherwise (Gibson, 1986; Marcus et al., 2016 Rotter et al., 2007). The supposed agnosticism with which “general auditory” theories deal with speech—that is, reducing it to an understanding of acoustic structure equivalent with its current spectral properties and separating it from the just-previous context–thus risks leaving “general auditory” accounts in the position of ignoring long-range events that might produce later spectral structure in the complete absence of sound, let alone the absence of speech. Assurances about perhaps a longer-term spectral average may fall flat in light of the present work's use of long-range linear surrogates. Indeed, an entirely speech-specific reason for admitting a broader notion of context in a theory of speech perception is that the context for coarticulation can be a future, not-yet-articulated, but anticipated phoneme (Maruthy et al., 2018). So, enforcing strict definitions of context as just-previous past risks ensuring an inability to explain anticipatory coarticulation.

## Conclusions

For present purposes, we take the gestural-theoretic position of admitting a context with important structure at many different scales, and we propose that a multifractal model of speech signals might offer a way to characterize speech as a physiological product embedded within interactions across multiple scales of space and time. Generally speaking, acoustic signals produced for communicative purposes exhibit a fractal-like hierarchical organization across time scales, and they do so regardless of whether they are human speech (Kello et al., 2017). Furthermore, if gestures are what listeners respond to, gestures are expressions of a movement system widely characterized by multifractal structure (Turvey and Fonseca, 2014). Unsurprisingly, gestures are themselves multifractal (Ashenfelter et al., 2009). Some of this multifractal structure comes from the fact that the movements system can pick up multifractal structure from a task environment (Stephen and Dixon, 2011). Furthermore, multifractality appears to spread easily from one part of the movement system to another (Carver et al., 2017). Hence, if gestures are central to speech perception, then any structure in gestures or their consequences for a listener's comprehension of speech should depend on multifractal structure. Our aim in the present work is to situate the gestural account of speech perception on multifractal foundations.

### Listening is sensitive to action

Section Effects of Context Multifractality on Perceptual Responses (Proportion of “GA” Selections) and Response Times (RT) thus makes the case that multifractality of context sounds may provide a key medium through which the speech signal communicates gestures and not just auditory information to the listener. The fact that multifractality predicted response behavior without qualification by context suggests that multifractality may actually provide even more general a framework in which to understand coarticulation than even the proposed “general-auditory” framework had. Crucially, multifractality addresses just those turbulent aspects of speech stream dynamics, physiology, as well as of social-cognitive contexts framing speech. The extremely specific case of turbulent structure of speech exemplifies what is generally typical of all acoustic distributions and speech behaviors in environments or surroundings where listeners draw meaning from sound stimuli speech or otherwise. Hence, multifractality might generalize a great deal of the contextual overlap noted by gestural accounts of speech perception and operationalize it in neat logical terms where multifractality can address specifically nonlinear interactions across scales (Mandelbrot, 1974; Schertzer and Lovejoy, 1985; Ihlen and Vereijken, 2010; Turvey and Fonseca, 2014).

The work in section Effects of Context Multifractality on Perceptual Responses (Proportion of “GA” Selections) and Response Times (RT) offers a potentially intriguing statistical relationship between phonemic perception and the multifractal structure of context stimuli preceding the target phoneme. What it does not offer is a proposed mechanism. Without addressing this point, a reader might easily come to the conclusion that we might envision a latent cognitive or perceptual factor responsible for detecting multifractality in the context stimulus, calculating multifractal estimates, and deploying these calculated estimates in the motor planning of a response to complete the task. We offer this secondary analysis in section Effects of Context Multifractality on Mouse-Cursor Trajectories on the Way to Clicked Response to instead offer the secondary proposal that multifractality in the context stimuli resonates with the multifractal structure of the movements system. That is to say, despite seeming sometimes like the most passive perceptual modality in which we let sound wash over hair cells in the cochlea, auditory perception especially for speech may enlist the movement system.

### Action in response to listening

Perhaps multifractality of all context sounds impress themselves not just on speech-perceptual responses but, more broadly, on the movements implicated in the decision process and behaviors supporting the perceptual response. This point here thus leads us to the strange but tantalizing possibility that the perception of speech enlists the movement system in ways that undermine the seemingly passive reception of speech sounds. Taken together with recent evidence that prosodic sing-song structure of spoken communication supports comprehension (Kelty-Stephen et al., 2018) and that song-like vocalizations exhibit multifractality due to nonlinear sources (Roeske et al., 2018), present results may extend to long-range speech comprehension.

We align the present work with a growing body of evidence and theory suggesting that the absorption of multifractal fluctuations by the movement system is a generic case of complexity matching in which the coupling of two systems occasions a sharing of multifractal fluctuations (Delignières et al., 2016; Almurad et al., 2017; Mahmoodi et al., 2017). Neither would it be the first time that speech perception has been understood as a sort of complexity matching. Abney et al. (2014) recorded individual speakers' speech sounds during an entire dyadic conversation and they assessed the monofractal structure for the individual speakers. They found that affiliative conversations led speakers to match one another's fractal patternings of speech. They interpreted this finding as evidence of complexity matching, specifically raising the possibility that systems poised to agree and to share information would be more likely to tailor their fractal patternings to one another. This intriguing work proposed to pool the many scales of variability in speech production from the phonemic scale of individual voicings of sounds to the discourse scale of an entire 10 min conversation.

The present work aims to set the notion of complexity matching atop the extremely fine time scale of the 375 ms of context-sound stimuli. Whereas a 10 min conversation affords room for variability in speech sounds to absorb a variety of emotional, cognitive, and social factors, the 375 ms of context for a subsequent phoneme leaves remarkably little room for anything but the multifractal geometry. 375 ms is a razor's edge on which we might try to balance the proposed factor of multifractal structure and to launch it into the movements system of a listener poised to decide the target phoneme following the 375-ms context.

The movement system of the listener echoes broadly with the multifractality of context sounds. That is, the multifractality is effective not just in quantifying a variable that might address key factors underlying speech perception. It may be providing a modality-general geometrical measure of stimulus energy that holds a common currency with the geometry of the movement system. As noted above, the progress on “complexity matching” has progressed mostly with regard movement systems whether between or within them (e.g., Kelty-Stephen and Dixon, 2014). So far, the movement system appears in “motor theories” of speech perception (Liberman and Mattingly, 1985; Galantucci et al., 2006), but gestural theories had once rejected the idea that the movement system is involved in speech perception (Fowler, 1996) but has recently reconsidered this position (Fowler, 2008) in light of more recent evidence (Fadiga et al., 2002). And research by gestural-theorists is certainly cognizant that speech perception occurs in an auditory scene and has found effects of spatial separation (Viswanathan et al., 2016). Multifractal may allow gestural theories to explain how movement systems support the speech-perceptual process.

## Author contributions

RW contributed to experimental design, collected all human subjects data, contributed to data analysis as well as to composition of the manuscript. DK-S contributed to experimental design, to data collection and to composition of the manuscript.

### Conflict of interest statement

The authors declare that the research was conducted in the absence of any commercial or financial relationships that could be construed as a potential conflict of interest.

## Supplementary material

Supplementary Figure 1 Model predictions representing the effects of multifractality W_MF_ as well as its interactions with Precursor, with entropy measures ψ and ξ on the mouse-tracking measure of maximum displacement (MD). These ψ × ξ × W_MF_×Precursor interaction effects on MD manifested differently across all context conditions: in real-speech context (left panel), in simulated-speech context (top right panel), and the tone context (bottom right panel). In all panels, interaction effects appear in solid lines (for Precursor [al]) or in dashed lines (for Precursor [aɹ]), in black (for low ψ) or in gray (for high ψ), and with circle markers (for high ξ) or without circle markers (for low ξ).

Supplementary Figure 2 Model predictions representing the effects of multifractal-based nonlinear estimate t_MF_ as well as its interactions with Precursor, with entropy measures ψ and ξ on the mouse-tracking measure of maximum displacement (MD). These ψ × ξ × t_MF_×Precursor interaction effects on MD manifested differently across all context conditions: in real-speech context (left panel), in simulated-speech context (top right panel), and the tone context (bottom right panel). In all panels, interaction effects appear in solid lines (for Precursor [al]) or in dashed lines (for Precursor [aɹ]), in black (for low ψ) or in gray (for high ψ), and with circle markers (for high ξ) or without circle markers (for low ξ).

Supplementary Figure 3 Model predictions representing the effects of multifractality W_MF_ × t_MF_ as well as its interactions with Precursor, with entropy measures ψ and ξ on the mouse-tracking measure of area under the curve (AUC). These ψ × ξ × W_MF_ × t_MF_ × Precursor interaction effects on AUC manifested differently across all context conditions: in real-speech context (left panel), in simulated-speech context (top right panel), and the tone context (bottom right panel). In all panels, interaction effects appear in solid lines (for Precursor [al]) or in dashed lines (for Precursor [aɹ]), in black (for low ψ) or in gray (for high ψ), and with circle markers (for high ξ) or without circle markers (for low ξ).

Supplementary Figure 4 Model predictions representing the effects of multifractality W_MF_ and its interactions with slow entropy ψ × W_MF_, with fast entropy ξ × W_MF_, and with precursor Precursor × W_MF_ on x-position flips. Panels show predictions specific to real-speech contexts (top left and in solid black), simulated-speech contexts (top right and in dashed black), and tone contexts (bottom right and in solid gray). Predictions for Precursor [al] appear as filled bars whereas predictions for Precursor [aɹ] appears empty bars. To show the range of multifractal properties in the simulated-speech cases, predictions appears for simulated-speech contexts with higher W_MF_ and higher t_MF_ appearing with higher-frequency dashes composing the borders and the filling of the bars.

Supplementary Table 1 All coefficients from logistic regression predicting “GA” vs. “DA” without any block or trial effects.

Supplementary Table 2 All coefficients from Poisson regression predicting cumulative “GA” responses without any block or trial effects.

Supplementary Table 3 All coefficients from Poisson regression predicting cumulative “GA” responses with block or trial effects.

Supplementary Table 4 Significant effects (*p* < 0.05) from linear regression of trial-by-trial maximum displacement (MD).

Supplementary Table 5 Significant effects (*p* < 0.05) from linear regression of trial-by-trial area under the curve (AUC).

Supplementary Table 6 Coefficients from Poisson regression of trial-by-trial x-position flips.
