# Supplementary figures and images for: Bringing the Nonlinearity of the Movement System to Gestural Theories of Language Use: Multifractal Structure of Spoken English Supports the Compensation for Coarticulation in Human Speech Perception
Source: Front Physiol. 2018 Sep 3;9:1152. doi: 10.3389/fphys.2018.01152 (PMC6129613; doi:10.3389/fphys.2018.01152)

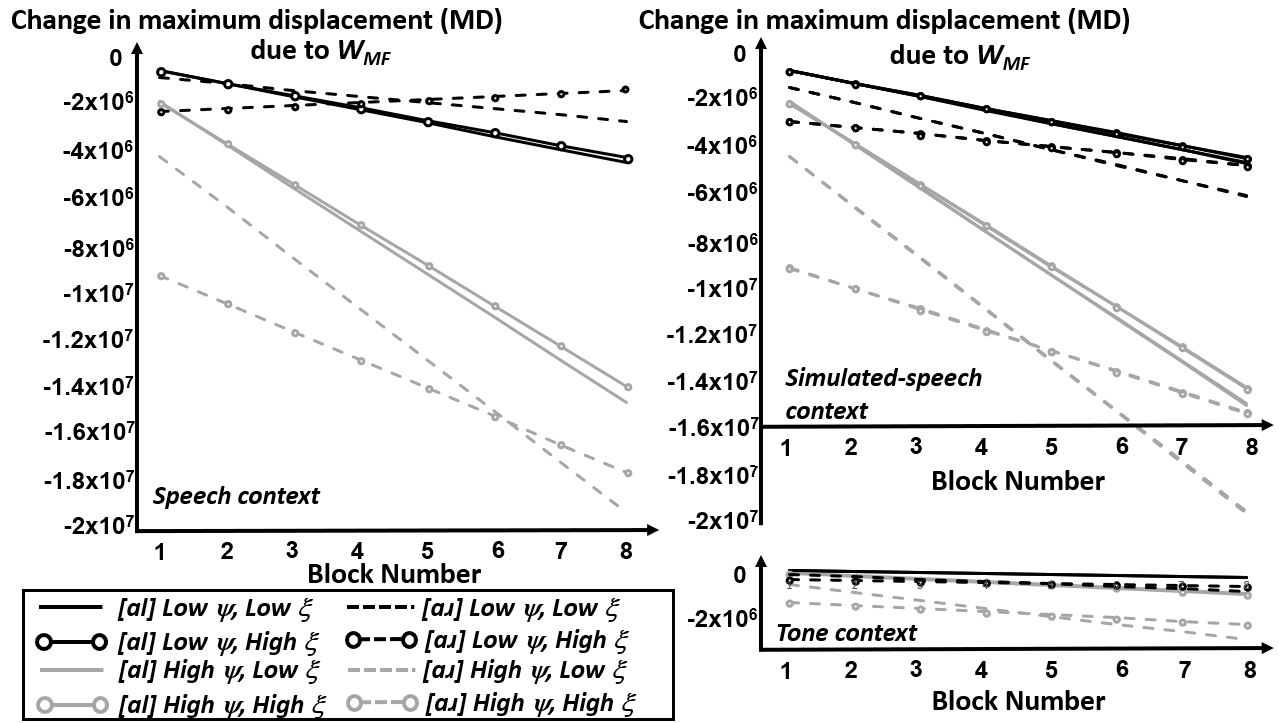

Supplement: Supplementary Figure 1 — Model predictions representing the effects of multifractality WMF as well as its interactions with Precursor, with entropy measures ψ and ξ on the mouse-tracking measure of maximum displacement (MD). These ψ × ξ × WMF×Precursor interaction effects on MD manifested differently across all context conditions: in real-speech context (left panel), in simulated-speech context (top right panel), and the tone context (bottom right panel). In all panels, interaction effects appear in solid lines (for Precursor [al]) or in dashed lines (for Precursor [aɹ]), in black (for low ψ) or in gray (for high ψ), and with circle markers (for high ξ) or without circle markers (for low ξ). [file Image_1.JPEG]

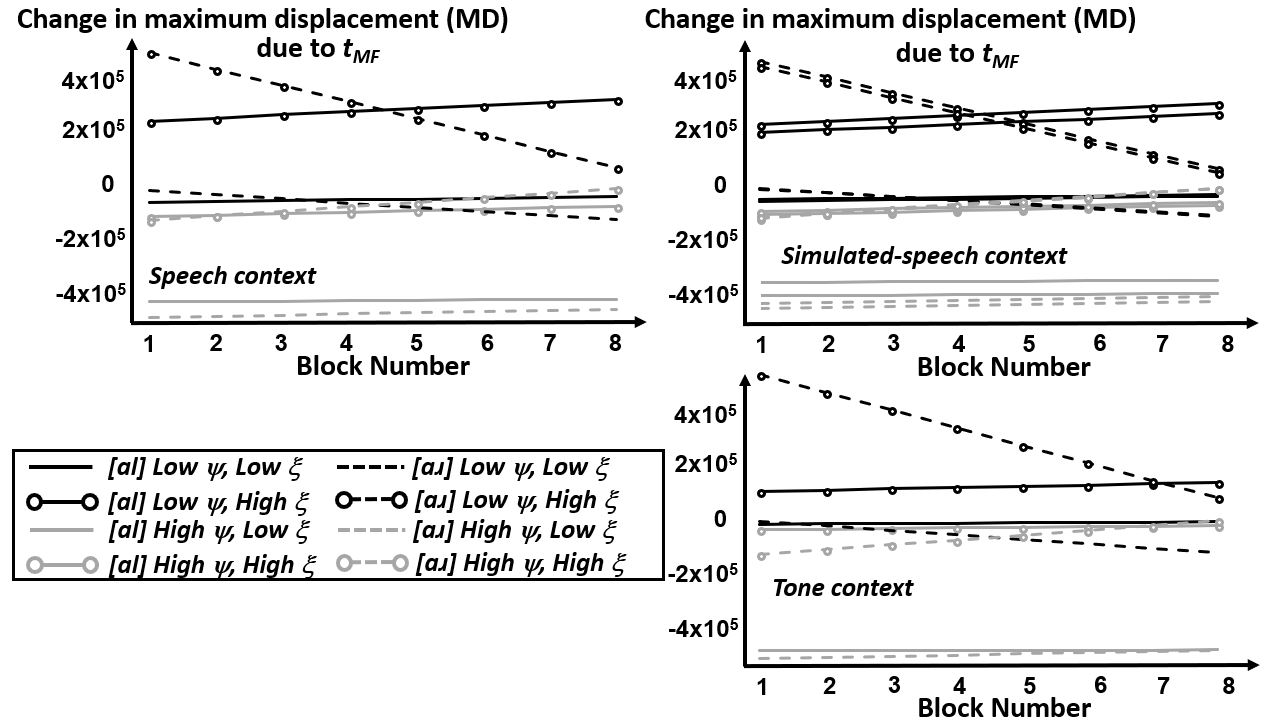

Supplement: Supplementary Figure 2 — Model predictions representing the effects of multifractal-based nonlinear estimate tMF as well as its interactions with Precursor, with entropy measures ψ and ξ on the mouse-tracking measure of maximum displacement (MD). These ψ × ξ × tMF×Precursor interaction effects on MD manifested differently across all context conditions: in real-speech context (left panel), in simulated-speech context (top right panel), and the tone context (bottom right panel). In all panels, interaction effects appear in solid lines (for Precursor [al]) or in dashed lines (for Precursor [aɹ]), in black (for low ψ) or in gray (for high ψ), and with circle markers (for high ξ) or without circle markers (for low ξ). [file Image_2.JPEG]

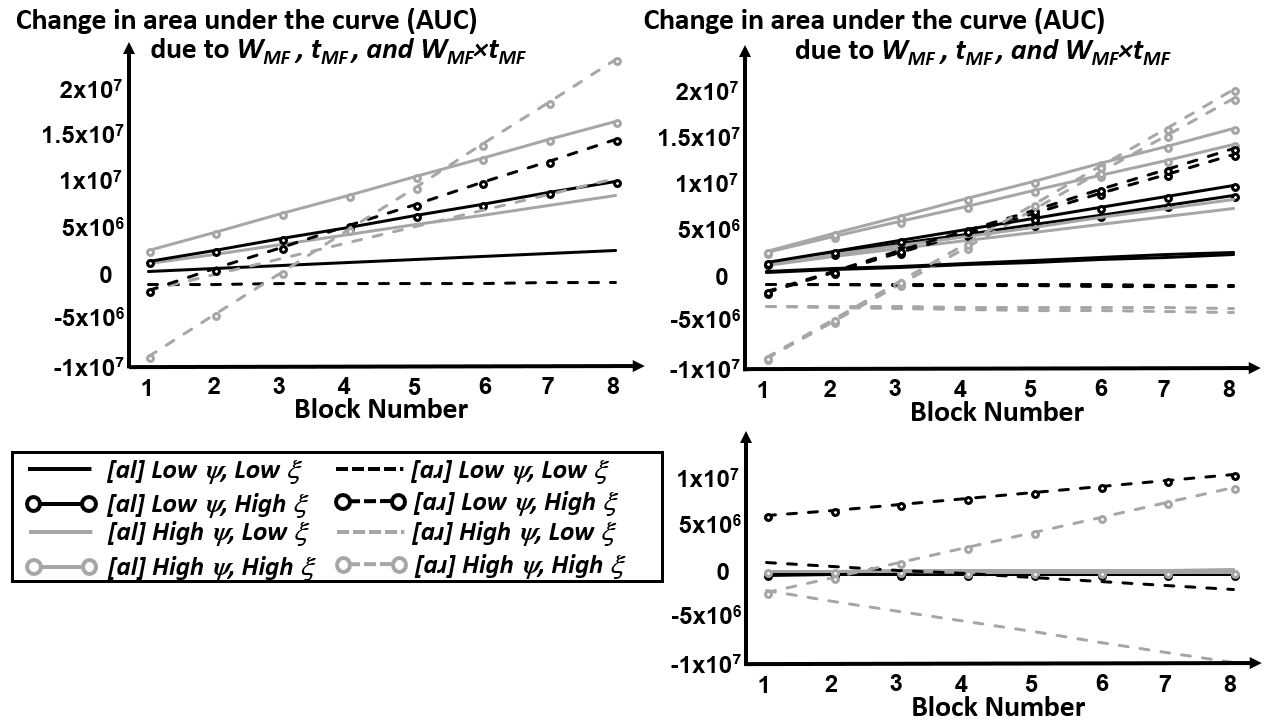

Supplement: Supplementary Figure 3 — Model predictions representing the effects of multifractality WMF × tMF as well as its interactions with Precursor, with entropy measures ψ and ξ on the mouse-tracking measure of area under the curve (AUC). These ψ × ξ × WMF × tMF × Precursor interaction effects on AUC manifested differently across all context conditions: in real-speech context (left panel), in simulated-speech context (top right panel), and the tone context (bottom right panel). In all panels, interaction effects appear in solid lines (for Precursor [al]) or in dashed lines (for Precursor [aɹ]), in black (for low ψ) or in gray (for high ψ), and with circle markers (for high ξ) or without circle markers (for low ξ). [file Image_3.JPEG]

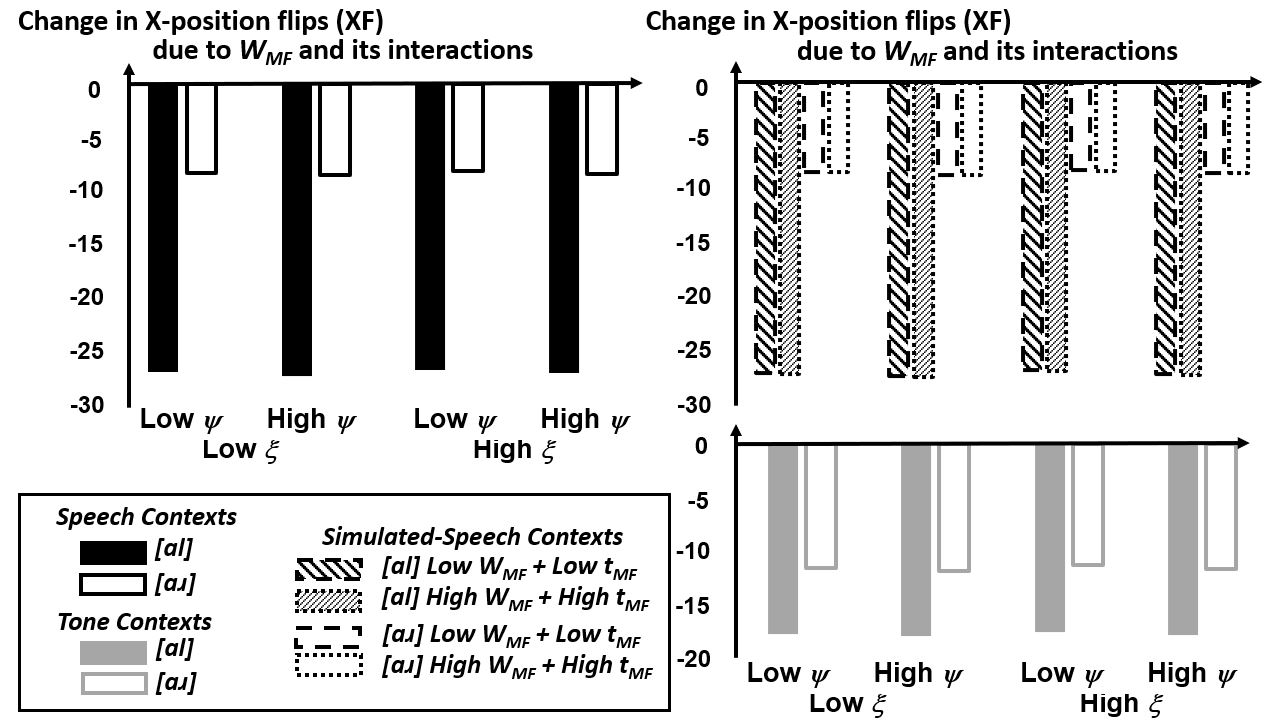

Supplement: Supplementary Figure 4 — Model predictions representing the effects of multifractality WMF and its interactions with slow entropy ψ × WMF, with fast entropy ξ × WMF, and with precursor Precursor × WMF on x-position flips. Panels show predictions specific to real-speech contexts (top left and in solid black), simulated-speech contexts (top right and in dashed black), and tone contexts (bottom right and in solid gray). Predictions for Precursor [al] appear as filled bars whereas predictions for Precursor [aɹ] appears empty bars. To show the range of multifractal properties in the simulated-speech cases, predictions appears for simulated-speech contexts with higher WMF and higher tMF appearing with higher-frequency dashes composing the borders and the filling of the bars. [file Image_4.jpg]
