# Supplementary material for: Bringing the Nonlinearity of the Movement System to Gestural Theories of Language Use: Multifractal Structure of Spoken English Supports the Compensation for Coarticulation in Human Speech Perception
Source: Front Physiol. 2018 Sep 3;9:1152. doi: 10.3389/fphys.2018.01152 (PMC6129613; doi:10.3389/fphys.2018.01152)
Supplement: Supplementary Table 1 — All coefficients from logistic regression predicting “GA” vs. “DA” without any block or trial effects. [file Table_1.DOCX]

Supplementary Material

Bringing the nonlinearity of the movement system to gestural theories of language use: Multifractal structure of spoken English supports the compensation for coarticulation in human speech perception

Rachel M. Ward, Damian G. Kelty-Stephen*

*** Correspondence:** Damian G. Kelty-Stephen, foovian@gmail.com

**Supplementary Table 1.** All coefficients from logistic regression predicting “GA” vs. “DA” without any block or trial effects

| Predictor | *B* | *SE* | *p* |
| --- | --- | --- | --- |
| Context(RealSpeech[RS]) | 4.47 | .27 | < .0001 |
| Context(Tone) | .42 | .36 | .24 |
| Context(SimulatedSpeech[SS]) | .10 | .36 | .78 |
| **Linear(Step)** | **-.78** | **.02** | **< .0001** |
| **Precursor** | **-.56** | **.12** | **< .0001** |
| Precursor×Context(Tone) | -.06 | .17 | .70 |
| Precursor×Context(SS) | -.29 | .17 | .09 |
