# Supplementary material for: Bringing the Nonlinearity of the Movement System to Gestural Theories of Language Use: Multifractal Structure of Spoken English Supports the Compensation for Coarticulation in Human Speech Perception
Source: Front Physiol. 2018 Sep 3;9:1152. doi: 10.3389/fphys.2018.01152 (PMC6129613; doi:10.3389/fphys.2018.01152)
Supplement: Supplementary Table 2 — All coefficients from Poisson regression predicting cumulative “GA” responses without any block or trial effects. [file Table_2.DOCX]

Supplementary Material

Bringing the nonlinearity of the movement system to gestural theories of language use: Multifractal structure of spoken English supports the compensation for coarticulation in human speech perception

Rachel M. Ward, Damian G. Kelty-Stephen*

*** Correspondence:** Damian G. Kelty-Stephen, foovian@gmail.com

**Supplementary Table 2**. All coefficients from Poisson regression predicting cumulative “GA” responses without any block or trial effects

| Predictor | *B* | *SE* | *p* |
| --- | --- | --- | --- |
| Intercept | 3.73 | .10 | < .0001 |
| Context(Tone) | .07 | .14 | .64 |
| Context(SimulatedSpeech[SS]) | -.26 | .14 | .07 |
| **Linear(Step)** | **-.0039** | **.0006** | **< .0001** |
| Precursor | -.0047 | .0063 | .46 |
| Precursor×Context(Tone) | .0054 | .0087 | .54 |
| Precursor×Context(SS) | .0056 | .0092 | .54 |
