# Supplementary material for: Bringing the Nonlinearity of the Movement System to Gestural Theories of Language Use: Multifractal Structure of Spoken English Supports the Compensation for Coarticulation in Human Speech Perception
Source: Front Physiol. 2018 Sep 3;9:1152. doi: 10.3389/fphys.2018.01152 (PMC6129613; doi:10.3389/fphys.2018.01152)
Supplement: Supplementary Table 3 — All coefficients from Poisson regression predicting cumulative “GA” responses with block or trial effects. [file Table_3.DOCX]

Supplementary Material

Bringing the nonlinearity of the movement system to gestural theories of language use: Multifractal structure of spoken English supports the compensation for coarticulation in human speech perception

Rachel M. Ward, Damian G. Kelty-Stephen*

*** Correspondence:** Damian G. Kelty-Stephen, foovian@gmail.com

**Supplementary Table 3**. All coefficients from Poisson regression predicting cumulative “GA” responses with block or trial effects

| Predictor | *B* | *SE* | *p* |
| --- | --- | --- | --- |
| Intercept | 1.95 | .10 | < .0001 |
| Context(Tone) | .09 | .14 | .53 |
| Context(SimulatedSpeech[SS]) | -.36 | .14 | < .05 |
| Linear(Step) | -.0071 | .0009 | < .0001 |
| Precursor | -.0012 | .0063 | .85 |
| Precursor×Context(Tone) | .0011 | .0087 | .90 |
| Precursor×Context(SS) | -.0002 | .0092 | .98 |
| *Over-time effects: Block number, trial number within block, and interactions* | | | |
| **Trial** | **.03** | **.0009** | **< .0001** |
| **Block** | **.31** | **.0027** | **< .0001** |
| **Block×Trial** | **-.0035** | **.0001** | **< .0001** |
| Block×Context(Tone) | -.0030 | .0021 | .16 |
| **Block×Context(SS)** | **.02** | **.0023** | **< .0001** |
| **Block×Linear(Step)** | **.0010** | **.0003** | **< .001** |
