# Supplementary material for: Bringing the Nonlinearity of the Movement System to Gestural Theories of Language Use: Multifractal Structure of Spoken English Supports the Compensation for Coarticulation in Human Speech Perception
Source: Front Physiol. 2018 Sep 3;9:1152. doi: 10.3389/fphys.2018.01152 (PMC6129613; doi:10.3389/fphys.2018.01152)
Supplement: Supplementary Table 4 — Significant effects (p < 0.05) from linear regression of trial-by-trial maximum displacement (MD). [file Table_4.DOCX]

Supplementary Material

Bringing the nonlinearity of the movement system to gestural theories of language use: Multifractal structure of spoken English supports the compensation for coarticulation in human speech perception

Rachel M. Ward, Damian G. Kelty-Stephen*

*** Correspondence:** Damian G. Kelty-Stephen, foovian@gmail.com

**Supplementary Table 4.** Significant effects (*p* < .05) from linear regression of trial-by-trial maximum displacement (MD)

| Predictor | *B* | *SE* |
| --- | --- | --- |
| *Interactions of multifractal-spectrum width W_MF_ with Step, Precursor, and Context interacting with Entropy Measures ψ and ξ in Mouse Tracking* | | |
| ψ×ξ×W_MF_ | 7.22×10^7^ | 3.27×10^7^ |
| ψ×W_MF_×Precursor×Block | -1.10×10^7^ | 5.48×10^6^ |
| ψ×ξ×W_MF_×Block | -1.41×10^7^ | 6.64×10^6^ |
| ψ×ξ×W_MF_×Precursor | -7.38×10^7^ | 2.20×10^7^ |
| ψ×ξ×W_MF_×Precursor×Block | 1.51×10^7^ | 4.69×10^7^ |
| ψ×ξ×W_MF_×Linear(Step)×Precursor×Block | -8.87×10^8^ | 3.46×10^8^ |
| *Interactions of nonlinearity t_MF_ with Step, Precursor, and Context interacting with Entropy Measures ψ and ξ in Mouse Tracking* | | |
| ξ×t_MF_ | 3.17×10^4^ | 1.09×10^4^ |
| ξ×t_MF_×Block | -8.50×10^3^ | 2.29×10^3^ |
| ξ×t_MF_×Precursor | -2.86×10^4^ | 1.03×10^4^ |
| ψ×ξ×t_MF_ | -2.77×10^4^ | 6.62×10^3^ |
| ξ×t_MF_×Precursor×Block | 7.40×10^3^ | 2.16×10^3^ |
| ψ×ξ×t_MF_×Block | 7.55×10^3^ | 1.43×10^3^ |
| ψ×ξ×t_MF_×Precursor | 2.51×10^4^ | 6.21×10^3^ |
| ψ×ξ×t_MF_×Precursor×Block | -6.97×10^3^ | 1.36×10^3^ |
| *Effects of Step, Precursor, and Context interacting with Entropy Measures ψ and ξ in Mouse Tracking*  *(omitting multifractal measures [W_MF_ and t_MF_] and counterbalancing [CB] of screen position of “GA”)* | | |
| ψ×ξ×Precursor | 7.85×10^6^ | 2.71×10^6^ |
| ξ×Precursor×Context(SS)×Block | -2.23×10^4^ | 8.52×10^3^ |
| ψ×ξ×Precursor×Context(Tone) | -6.35×10^6^ | 2.42×10^6^ |
| ψ×ξ×Precursor×Block | -1.61×10^6^ | 5.64×10^5^ |
| ψ×Linear(Step)×Context(Tone)×Block×Trial | -9.17×10^4^ | 4.65×10^4^ |
| ψ×ξ×Linear(Step)×Precursor×Block | 8.80×10^7^ | 4.19×10^7^ |
| ψ×ξ×Precursor×Context(Tone)×Block | 1.27×10^6^ | 4.98×10^5^ |
| ψ×ξ×Precursor×Context(SS)×Block | 1.21×10^4^ | 4.76×10^3^ |
| ψ×ξ×Linear(Step)×Precursor×Context(Tone)×Block | -7.59×10^7^ | 3.79×10^7^ |
| ψ×ξ×Linear(Step)×Precursor×Block×Trial | 4.88×10^4^ | 2.10×10^4^ |
| ψ×ξ×Linear(Step)×Precursor×Context(Tone)×Block×Trial | -8.87×10^8^ | 3.46×10^8^ |
| *Effects due to counterbalanced screen position of “GA” versus “DA”* | | |
| CB×ψ | 8.50×10^4^ | 3.09×10^4^ |
| CB×ψ×ξ | -7.01×10^4^ | 3.49×10^4^ |
| CB×ψ×Trial | -9.05×10^3^ | 2.50×10^3^ |
| CB×ψ×Precursor | -4.07×10^4^ | 1.99×10^4^ |
| CB×ψ×Context(Tone) | -1.21×10^5^ | 4.66×10^4^ |
| CB×ψ×Context(SS) | -1.49×10^5^ | 4.53×10^4^ |
| CB×ψ×Block×Trial | 1.64×10^3^ | 5.15×10^2^ |
| CB×ψ×Precursor×Trial | 4.90×10^3^ | 1.62×10^3^ |
| CB×ψ×Quadratic(Step)×Block | -1.55×10^6^ | 5.35×10^5^ |
| CB×ξ×Quadratic(Step)×Block | 2.02×10^6^ | 9.82×10^5^ |
| CB×ξ×Precursor×Trial | -6.16×10^3^ | 2.68×10^3^ |
| CB×ψ×ξ×Context(SS) | 9.58×10^4^ | 4.50×10^4^ |
| CB×ψ×Precursor×Context(Tone) | 6.17×10^4^ | 2.90×10^4^ |
| CB×ψ×Precursor×Context(SS) | 7.88×10^4^ | 2.95×10^4^ |
| CB×ψ×Context(Tone)×Block | 2.11×10^4^ | 1.01×10^4^ |
| CB×ψ×Context(SS)×Block | 2.37×10^4^ | 9.95×10^3^ |
| CB×ψ×Context(Tone)×Trial | 1.20×10^4^ | 3.62×10^3^ |
| CB×ψ×Context(SS)×Trial | 7.17×10^3^ | 3.59×10^3^ |
| CB×ψ×Linear(Step)×Context(SS) | 1.20×10^7^ | 4.13×10^6^ |
| CB×ψ×Linear(Step)×Precursor×Context(SS) | -7.53×10^6^ | 2.73×10^6^ |
| CB×ψ×Linear(Step)×Context(SS)×Block | -2.75×10^6^ | 9.23×10^5^ |
| CB×ψ×Linear(Step)×Context(SS)×Trial | -9.09×10^5^ | 3.21×10^5^ |
| CB×ψ×Quadratic(Step)×Precursor×Block | 9.21×10^5^ | 3.64×10^5^ |
| CB×ψ×Quadratic(Step)×Context(SS)×Block | 2.25×10^6^ | 8.95×10^5^ |
| CB×ξ×Quadratic(Step)×Block×Trial | -1.57×10^5^ | 7.76×10^4^ |
| CB×ξ×Context(Tone)×Block×Trial | 2.60×10^3^ | 1.17×10^3^ |
| CB×ψ×Precursor×Block×Trial | -8.49×10^2^ | 3.24×10^2^ |
| CB×ψ×Precursor×Context(Tone)×Trial | -6.51×10^3^ | 2.27×10^3^ |
| CB×ψ×Context(Tone)×Block×Trial | -2.70×10^3^ | 7.68×10^2^ |
| CB×ψ×Context(SS)×Block×Trial | -1.76×10^3^ | 7.87×10^2^ |
| CB×ξ×Precursor×Block×Trial | 1.39×10^3^ | 5.27×10^2^ |
| CB×ψ×Precursor×Context(Tone)×Block×Trial | 1.48×10^3^ | 4.77×10^2^ |
| CB×ψ×Linear(Step)×Context(SS)×Block×Trial | 2.28×10^5^ | 7.21×10^4^ |
| CB×ψ×Linear(Step)×Precursor×Context(SS)×Block | 1.86×10^6^ | 6.20×10^5^ |
| CB×ψ×Linear(Step)×Precursor×Context(SS)×Trial | 5.85×10^5^ | 2.12×10^5^ |
| CB×ψ×Quadratic(Step)×Precursor×Context(SS)×Block | -1.19×10^6^ | 5.90×10^5^ |
| CB×ψ×Quadratic(Step)×Context(SS)×Block×Trial | -1.72×10^5^ | 6.93×10^4^ |
| CB×ξ×Precursor×Context(Tone)×Block×Trial | -2.21×10^3^ | 7.37×10^2^ |
| CB×ψ×Linear(Step)×Precursor×Context(SS)×Block×Trial | -1.61×10^6^ | 7.99×10^5^ |
| CB×ψ×Quadratic(Step)×Precursor×Context(SS)×Block | -1.53×10^5^ | 4.90×10^4^ |
| CB×ψ×ξ×Quadratic(Step)×Precursor×Context(SS) | -5.19×10^6^ | 2.60×10^6^ |
| CB×ψ×ξ×Quadratic(Step)×Context(SS)×Block×Trial | 1.38×10^5^ | 6.33×10^4^ |
| CB×ψ×ξ×Quadratic(Step)×Precursor×Context(SS)×Block | 1.44×10^6^ | 5.41×10^5^ |
| CB×ψ×ξ×Quadratic(Step)×Precursor×Context(SS)×Trial | 4.13×10^5^ | 2.03×10^5^ |
| CB×ψ×ξ×Quadratic(Step)×Precursor×Context(SS)×Precursor  ×Block×Trial | -1.32×10^5^ | 4.27×10^4^ |
