# Supplementary material for: Bringing the Nonlinearity of the Movement System to Gestural Theories of Language Use: Multifractal Structure of Spoken English Supports the Compensation for Coarticulation in Human Speech Perception
Source: Front Physiol. 2018 Sep 3;9:1152. doi: 10.3389/fphys.2018.01152 (PMC6129613; doi:10.3389/fphys.2018.01152)
Supplement: Supplementary Table 5 — Significant effects (p < 0.05) from linear regression of trial-by-trial area under the curve (AUC). [file Table_5.DOCX]

Supplementary Material

Bringing the nonlinearity of the movement system to gestural theories of language use: Multifractal structure of spoken English supports the compensation for coarticulation in human speech perception

Rachel M. Ward, Damian G. Kelty-Stephen*

*** Correspondence:** Damian G. Kelty-Stephen, foovian@gmail.com

**Supplementary Table 5.** Significant effects (*p* < .05) from linear regression of trial-by-trial area under the curve (AUC)

| Predictor | *B* | *SE* |
| --- | --- | --- |
| *Interactions of multifractal-spectrum width W_MF_ with Step, Precursor, and Context interacting with Entropy Measures ψ and ξ in Mouse Tracking* | | |
| ψ×ξ×W_MF_ | 7.60×10^6^ | 3.64×10^6^ |
| ψ×ξ×W_MF_×Quadratic(Step) | -3.61×10^8^ | 9.96×10^7^ |
| ψ×ξ×W_MF_×Block | -2.20×10^6^ | 7.69×10^5^ |
| ψ×W_MF_×Quadratic(Step)×Block | -6.31×10^7^ | 2.98×10^7^ |
| ξ×W_MF_×Quadratic(Step)×Trial | -3.68×10^7^ | 1.54×10^7^ |
| ψ×W_MF_×Quadratic(Step)×Precursor×Block | 6.29×10^7^ | 2.98×10^7^ |
| ψ×W_MF_×Quadratic(Step)×Block×Trial | 5.23×10^6^ | 2.31×10^6^ |
| ξ×W_MF_×Quadratic(Step)×Precursor×Trial | 3.57×10^7^ | 1.54×10^7^ |
| ψ×ξ×W_MF_×Quadratic(Step)×Precursor | 3.52×10^8^ | 9.93×10^7^ |
| ψ×ξ×W_MF_×Quadratic(Step)×Block | 9.56×10^7^ | 2.27×10^7^ |
| ψ×ξ×W_MF_×Quadratic(Step)×Trial | 3.23×10^7^ | 8.33×10^6^ |
| ψ×ξ×W_MF_×Precursor×Block | 2.12×10^6^ | 7.51×10^5^ |
| ψ×W_MF_×Quadratic(Step)×Precursor×Block×Trial | -5.23×10^6^ | 2.31×10^6^ |
| ψ×ξ×W_MF_×Quadratic(Step)×Precursor×Block | -9.41×10^7^ | 2.27×10^7^ |
| ψ×ξ×W_MF_×Quadratic(Step)×Precursor×Trial | -3.16×10^7^ | 8.31×10^6^ |
| ψ×ξ×W_MF_×Quadratic(Step)×Block×Trial | -8.19×10^6^ | 1.99×10^5^ |
| ψ×ξ×W_MF_×Quadratic(Step)×Precursor×Block×Trial | 8.06×10^6^ | 1.99×10^6^ |
| *Interactions of nonlinearity t_MF_ with Step, Precursor, and Context interacting with Entropy Measures ψ and ξ in Mouse Tracking* | | |
| ψ×ξ×t_MF_ | -5.40×10^4^ | 2.58×10^4^ |
| ξ×t_MF_×Quadratic(Step) | -2.61×10^6^ | 1.13×10^6^ |
| ψ×t_MF_×Quadratic(Step)×Block | 3.69×10^5^ | 1.75×10^5^ |
| ξ×t_MF_×Quadratic(Step)×Precursor | 2.30×10^6^ | 1.09×10^6^ |
| ξ×t_MF_×Quadratic(Step)×Trial | 2.51×10^5^ | 9.15×10^4^ |
| ψ×ξ×t_MF_×Quadratic(Step) | 2.38×10^6^ | 5.99×10^5^ |
| ψ×ξ×t_MF_×Precursor | 4.72×10^4^ | 2.39×10^4^ |
| ψ×ξ×t_MF_×Block | 1.50×10^4^ | 5.38×10^3^ |
| ψ×t_MF_×Quadratic(Step)×Precursor×Block | -3.62×10^5^ | 1.70×10^5^ |
| ψ×t_MF_×Quadratic(Step)×Block×Trial | -3.02×10^4^ | 1.35×10^4^ |
| ξ×t_MF_×Quadratic(Step)×Precursor×Trial | -2.24×10^5^ | 8.85×10^4^ |
| ψ×ξ×t_MF_×Quadratic(Step)×Precursor | -2.17×10^6^ | 5.73×10^5^ |
| ψ×ξ×t_MF_×Quadratic(Step)×Block | -6.03×10^5^ | 1.34×10^5^ |
| ψ×ξ×t_MF_×Quadratic(Step)×Trial | -2.11×10^5^ | 4.98×10^4^ |
| ψ×ξ×t_MF_×Precursor×Block | -1.41×10^4^ | 5.06×10^3^ |
| ψ×t_MF_×Quadratic(Step)×Precursor×Block×Trial | 2.99×10^4^ | 1.32×10^4^ |
| ψ×ξ×t_MF_×Quadratic(Step)×Precursor×Block | 5.64×10^5^ | 1.30×10^5^ |
| ψ×ξ×t_MF_×Quadratic(Step)×Precursor×Trial | 1.93×10^5^ | 4.78×10^4^ |
| ψ×ξ×t_MF_×Quadratic(Step)×Block×Trial | 5.17×10^4^ | 1.17×10^4^ |
| ψ×ξ×t_MF_×Quadratic(Step)×Precursor×Block×Trial | -4.84×10^4^ | 1.14×10^4^ |
| *Interactions of W_MF_*×*t_MF_ with Step, Precursor, and Context interacting with Entropy Measures ψ and ξ in Mouse Tracking* | | |
| ψ×ξ×W_MF_×t_MF_ | 4.87×10^5^ | 2.40×10^5^ |
| ξ×W_MF_×t_MF_×Quadratic(Step) | 2.37×10^7^ | 1.08×10^7^ |
| ψ×W_MF_×t_MF_×Quadratic(Step)×Block | -3.57×10^6^ | 1.68×10^6^ |
| ξ×W_MF_×t_MF_×Quadratic(Step)×Precursor | -2.16×10^7^ | 1.06×10^7^ |
| ξ×W_MF_×t_MF_×Quadratic(Step)×Trial | -2.29×10^6^ | 8.75×10^5^ |
| ψ×ξ×W_MF_×t_MF_×Quadratic(Step) | -2.20×10^7^ | 5.68×10^6^ |
| ψ×ξ×W_MF_×t_MF_×Block | -1.41×10^5^ | 5.07×10^4^ |
| ψ×W_MF_×t_MF_×Quadratic(Step)×Precursor×Block | 3.53×10^6^ | 1.66×10^6^ |
| ψ×W_MF_×t_MF_×Quadratic(Step)×Block×Trial | 2.93×10^5^ | 1.30×10^5^ |
| ξ×W_MF_×t_MF_×Quadratic(Step)×Precursor×Trial | 2.11×10^6^ | 8.60×10^5^ |
| ψ×ξ×W_MF_×t_MF_×Quadratic(Step)×Precursor | 2.05×10^7^ | 5.56×10^6^ |
| ψ×ξ×W_MF_×t_MF_×Precursor×Block | 1.35×10^5^ | 4.91×10^4^ |
| ψ×ξ×W_MF_×t_MF_×Quadratic(Step)×Block | 5.66×10^6^ | 1.29×10^6^ |
| ψ×ξ×W_MF_×t_MF_×Quadratic(Step)×Trial | 1.96×10^6^ | 4.74×10^5^ |
| ψ×W_MF_×t_MF_×Quadratic(Step)×Precursor×Block×Trial | -2.92×10^5^ | 1.29×10^5^ |
| ψ×ξ×W_MF_×t_MF_×Quadratic(Step)×Precursor×Block | -5.40×10^6^ | 1.27×10^6^ |
| ψ×ξ×W_MF_×t_MF_×Quadratic(Step)×Precursor×Trial | -1.84×10^6^ | 4.64×10^5^ |
| ψ×ξ×W_MF_×t_MF_×Quadratic(Step)×Block×Trial | -4.85×10^5^ | 1.13×10^5^ |
| ψ×ξ×W_MF_×t_MF_×Quadratic(Step)×Precursor×Block×Trial | 4.63×10^5^ | 1.11×10^5^ |
| *Effects of Step, Precursor, and Context interacting with Entropy Measures ψ and ξ in Mouse Tracking*  *(omitting multifractal measures [W_MF_ and t_MF_] and counterbalancing [CB] of screen position of “GA”)* | | |
| ψ×ξ | -8.39×10^5^ | 3.87×10^5^ |
| ξ×Quadratic(Step) | -3.95×10^7^ | 1.95×10^7^ |
| ψ×Quadratic(Step)×Block | 6.46×10^6^ | 3.05×10^6^ |
| ξ×Quadratic(Step)×Trial | 3.86×10^6^ | 1.58×10^6^ |
| ψ×ξ×Quadratic(Step) | 3.77×10^7^ | 1.02×10^7^ |
| ψ×ξ×Precursor | 7.39×10^5^ | 3.64×10^5^ |
| ψ×ξ×Block | 2.32×10^5^ | 8.09×10^4^ |
| ψ×Quadratic(Step)×Precursor×Block | -6.42×10^6^ | 3.04×10^6^ |
| ψ×Quadratic(Step)×Block×Trial | -5.36×10^5^ | 2.36×10^5^ |
| ξ×Quadratic(Step)×Precursor×Trial | -3.70×10^6^ | 1.58×10^6^ |
| ψ×ξ×Quadratic(Step)×Precursor | -3.64×10^7^ | 1.02×10^7^ |
| ψ×ξ×Quadratic(Step)×Block | -9.92×10^6^ | 2.33×10^6^ |
| ψ×ξ×Quadratic(Step)×Trial | -3.37×10^6^ | 8.54×105 |
| ψ×ξ×Precursor×Block | -2.20×10^5^ | 7.72×10^4^ |
| ψ×Quadratic(Step)×Precursor×Block×Trial | 5.34×10^5^ | 2.36×10^5^ |
| ψ×ξ×Quadratic(Step)×Precursor×Block | 9.69×10^6^ | 2.32×10^6^ |
| ψ×ξ×Quadratic(Step)×Precursor×Trial | 3.26×10^6^ | 8.50×10^5^ |
| ψ×ξ×Quadratic(Step)×Block×Trial | 8.50×10^5^ | 2.04×10^5^ |
| ψ×ξ×Precursor×Context(SS)×Block | 4.18×10^1^ | 2.11×10^1^ |
| ψ×ξ×Quadratic(Step)×Precursor×Block×Trial | -8.30×10^5^ | 2.04×10^5^ |
| *Effects due to counterbalanced screen position of “GA” versus “DA”* | | |
| CB×ξ | 3.62×10^2^ | 1.39×10^2^ |
| CB×ψ×Block | 3.47×10^1^ | 1.61×10^1^ |
| CB×ξ×Precursor | -2.28×10^2^ | 8.81×10^1^ |
| CB×ξ×Block | -6.87×10^1^ | 2.71×10^1^ |
| CB×ψ×ξ×Context(SS) | 4.02×10^2^ | 1.20×10^2^ |
| CB×ψ×Quadratic(Step)×Block | -5.67×10^1^ | 2.42×10^1^ |
| CB×ξ×Precursor×Context(Tone) | 2.49×10^2^ | 1.22×10^2^ |
| CB×ξ×Precursor×Block | 3.72×10^1^ | 1.72×10^1^ |
| CB×ψ×Precursor×Context(Tone)×Block | 3.45×10^1^ | 1.55×10^1^ |
| CB×ψ×Precursor×Context(SS)×Block | -3.49×10^1^ | 1.73×10^1^ |
| CB×ξ×Precursor×Context(Tone)×Block | -4.91×10^1^ | 2.42×10^1^ |
| CB×ψ×ξ×Context(Tone)×Precursor | -1.47×10^2^ | 7.28×10^1^ |
| CB×ψ×ξ×Context(SS)×Precursor | -2.65×10^2^ | 7.59×10^1^ |
| CB×ψ×ξ×Context(SS)×Block | -6.34×10^1^ | 2.38×10^1^ |
| CB×ψ×ξ×Context(SS)×Precursor×Block | 4.51×10^1^ | 1.55×10^1^ |
