# Supplementary material for: Bringing the Nonlinearity of the Movement System to Gestural Theories of Language Use: Multifractal Structure of Spoken English Supports the Compensation for Coarticulation in Human Speech Perception
Source: Front Physiol. 2018 Sep 3;9:1152. doi: 10.3389/fphys.2018.01152 (PMC6129613; doi:10.3389/fphys.2018.01152)
Supplement: Supplementary Table 6 — Coefficients from Poisson regression of trial-by-trial x-position flips. [file Table_6.DOCX]

Supplementary Material

Bringing the nonlinearity of the movement system to gestural theories of language use: Multifractal structure of spoken English supports the compensation for coarticulation in human speech perception

Rachel M. Ward, Damian G. Kelty-Stephen*

*** Correspondence:** Damian G. Kelty-Stephen, foovian@gmail.com

**Supplementary Table 6.** Coefficients from Poisson regression of trial-by-trial x-position flips

| Predictor | *B* | *SE* | *p* |
| --- | --- | --- | --- |
| Intercept | 45.31 | 2.86 | < .0001 |
| ψ | 1.38 | .08 | < .0001 |
| ξ | -.64 | .09 | < .0001 |
| Linear(Step) | -8.92 | 1.43 | < .0001 |
| Quadratic(Step) | -2.85 | 1.40 | < .05 |
| Context(Tone) | -43.86 | 2.87 | < .0001 |
| Context(SS) | -.34 | .21 | .11 |
| Precursor | -18.81 | 1.60 | < .0001 |
| Precursor×Context(Tone) | 18.20 | 1.30 | < .0001 |
| Precursor×Context(SS) | .01 | .08 | .90 |
| W_MF_ | -287.80 | 15.50 | < .0001 |
| ψ×W_MF_ | -2.77 | .71 | < .001 |
| ξ×W_MF_ | 2.33 | .80 | < .01 |
| Precursor×W_MF_ | 103.10 | 21.06 | < .0001 |
| Block | -.11 | .01 | < .0001 |
| Trial | -.03 | .0051 | < .0001 |
| Block×Trial | .0042 | .0011 | < .001 |
| CB | -.09 | .14 | .51 |
